# A modular, open-source, slide-scanning microscope for diagnostic applications in resource-constrained settings

**DOI:** 10.1371/journal.pone.0194063

**Published:** 2018-03-15

**Authors:** Qiang Lu, Guanghui Liu, Chuanli Xiao, Chuanzhen Hu, Shiwu Zhang, Ronald X. Xu, Kaiqin Chu, Qianming Xu, Zachary J. Smith

**Affiliations:** 1 Department of Precision Machinery and Precision Instrumentation, University of Science and Technology of China, Hefei, Anhui, China; 2 School of Animal Science and Technology, Anhui Agricultural University, Hefei, Anhui, China; Texas A&M University, UNITED STATES

## Abstract

In this paper we report the development of a cost-effective, modular, open source, and fully automated slide-scanning microscope, composed entirely of easily available off-the-shelf parts, and capable of bright field and fluorescence modes. The automated X-Y stage is composed of two low-cost micrometer stages coupled to stepper motors operated in open-loop mode. The microscope is composed of a low-cost CMOS sensor and low-cost board lenses placed in a 4f configuration. The system has approximately 1 micron resolution, limited by the f/# of available board lenses. The microscope is compact, measuring just 25×25×30 cm, and has an absolute positioning accuracy of ±1 μm in the X and Y directions. A Z-stage enables autofocusing and imaging over large fields of view even on non-planar samples, and custom software enables automatic determination of sample boundaries and image mosaicking. We demonstrate the utility of our device through imaging of fluorescent- and transmission-dye stained blood and fecal smears containing human and animal parasites, as well as several prepared tissue samples. These results demonstrate image quality comparable to high-end commercial microscopes at a cost of less than US$400 for a bright-field system, with an extra US$100 needed for the fluorescence module.

## Introduction

As health care costs rise, there is an increasing interest in finding lower-cost methods of providing high-quality care. Substantial effort has been expended in developing portable and easy-to-use instrumentation that can replace costly and centralized medical instruments [1,2]. This is particularly relevant for rural [3] and low-resource settings [4], as well as combat settings and disaster areas [5], where centralized and well-equipped medical facilities may not be accessible to a large portion of the population. One critical component in pathologic diagnosis is computer-aided or manual examination of tissues and body fluids at microscopic resolution. One approach to automating this process is whole-slide imaging (WSI). In WSI, large areas of a sample are imaged and digitized for later review by pathologists either on-site or at remote locations. However, current adoption of WSI is low, and mostly used in niche applications [6]. In societies with highly developed medical infrastructure, the number of trained pathologists is large enough that manual examination of slides using costly conventional microscopes is a practical method for sample imaging and diagnosis. However, in resource-constrained settings, WSI may be coupled with telemedicine to alleviate the paucity of available experts, through remote evaluation of digitized images, via computerized, crowd-sourced [7], or expert analysis [8]. However, the utility of such systems depends upon their cost-effectiveness and simplicity. In order to see wide adoption in resource-constrained settings, an ideal system should be cheap enough to be widely deployed, and have the ability to be used by non-expert operators [9]. For example, a recent WSI telepathology system installed in Pakistan incurred serious delays in implementation due to the departure of a single pathologist [10].

Microscopic examination remains the gold-standard for many diseases. For example, while many parasitic diseases have convenient dipstick tests, these lack sensitivity and specificity [11]. Parasites and eggs have specific morphologies and staining characteristics that can be used to quickly identify disease and prescribe appropriate treatment [12], if a microscope and trained observer are available. However, the prohibitive cost of even manual microscopes has driven many researchers in the past several years to explore using low-cost consumer electronics to create cost-effective medical devices such as microscopes and colorimetric test readers [13–22]. Increasingly, these systems use advanced computational processing to allow virtually no trade-offs in performance compared to their conventional counterparts [23–26].

Less well explored is the ability of these complex systems to be operated by users without substantial experience [27]. This is critical as many of the emerging field trials of these cost-effective, consumer-electronics-enabled medical devices have shown substantially reduced diagnostic performance compared to standard methods [28–30]. Many of these systems, particularly those employing conventional optics, require significant skill to be easily operated. Manual movement of samples has been cited by Bogoch *et al*., for example, as one factor limiting diagnostic performance of a cell-phone-based microscope [28]. This is particularly critical for applications such as fecal or blood parasite screening where whole slides must be imaged in order to arrive at a definitive diagnosis. Thus, while motion control has the substantial drawback of requiring additional external power, it may provide a benefit in reducing the need for user expertise. However, complex motion control typically drastically increases the cost of the experimental system. Computational approaches such as lensless holography [14,24] or Fourier ptychographic microscopy [26] can capture large fields of view without the need for sample translation. However, these techniques are generally limited to transmission measurements and have difficulties measuring fluorescent samples at high resolution. Lens-free systems yield fluorescence images with poor resolution (~10 microns) and furthermore require a costly faceplate to be bonded to the image sensor [31]. Dong *et al*. have shown that by combining structured illumination combined with Fourier ptychographic reconstruction, the resolution of a standard widefield fluorescence system can be doubled. However this improvement is far short of the order of magnitude improvement provided by transmissive Fourier ptyochography [32]. Given the increasing reliance on fluorescent methods such as immunofluorescence and ELISA, there remains a need for low-cost combinations of robotic motion control with fluorescence-ready optical systems to realize truly widespread, cost-effective WSI microscopy.

Portable document scanners are one example of low-cost optical systems with embedded motion control. Several authors have reported their use in bright field and fluorescence microscopy applications, as summarized in a recent review by Göröcs and Ozcan [33]. These systems enable extremely large field of view images to be captured, but at modest resolutions of around 10 microns. Zheng and Ou *et al*. recently showed how using a high-quality photographic lens with high space-bandwidth product to project a sample onto a document scanner can yield large fields of view with approximately 1.5 micron resolution [34,35]. However, while this system does not require sample translation, it still requires automated motion control of the lens itself for focusing purposes. Further, because the document scanner images almost the entire usable field of view of the lens, the quality of the lens and scanner are critical, substantially increasing the system cost. Schaefer *et al*. have also demonstrated a low-cost microscope utilizing harvested components from CD players as motion controllers [36]. However, repurposing these components for use outside of their intended application leads to serious tradeoffs in precision and accuracy, and furthermore the instrument described in Ref. [36] requires extensive custom fabrication and engineering expertise to construct and utilize. Sharkey *et al*. have recently reported a carefully considered design for a monolithic 3D printed microscope stage with extremely high precision utilizing screw-driven flexure hinges [37]. Combined with low-cost motor control this system has the potential for highly accurate and repeatable sample positioning. However, the system must be completely custom fabricated and requires access to a reasonable quality 3D printer, as the print quality will directly translate into the stage’s performance. Further, due to the flexure hinge design, the travel range of this stage is necessarily limited to a few millimeters in X and Y to avoid permanent deformation of the hinges. Finally, Sung *et al*. utilize LEGO motors and bricks to perform low cost 1D translation of an optical testing system [38]. However, while the motors include their own LEGO-designed control system, obviating the need for an external computer, the motor and gear precisions are not high enough for microscopic focusing, as would be needed on a large-area sample where micron-scale flatness across the entire region of interest cannot be easily guaranteed.

Recently Campbell *et al*. demonstrated that off-the-shelf motors and controllers, such as those used in hobbyist robotics, operating in open-loop mode can be used to create submicron-precise motion control without the need for expensive closed-loop encoders [39]. This open-source motion control concept was dubbed by the authors as “OpenStage.” However, perhaps driven by the specific applications considered in that manuscript, the peripheral mechanics surrounding the motors and controllers still had a cost several orders of magnitude larger than the motors themselves.

In this manuscript, we build upon these earlier efforts, creating a microscope where highly precise and repeatable motion control is coupled to a cost-effective, consumer-electronics-enabled microscope to create a fully automated slide scanning microscope with both fluorescence and bright-field capabilities. One goal in constructing this system was to utilize only off-the-shelf components, such that, contrasting with other systems, absolutely no custom fabrication is needed at any step in the construction process. Thus, step-by-step instructions for purchase and construction, similar to assembling a LEGO toy, can be easily followed (see SI for further information). With these instructions, the system can be easily duplicated as a school or personal project, or by interested clinics or other health-care professionals. The system is modular by design, with the stage and scope able to operate entirely independently, with multiple imaging modes possible depending on user needs. Thus, the overall system can be easily modified or adjusted for particular applications. Due to using commoditized, off-the-shelf components, the cost is kept to around US$400 for the complete, automated microscope system.

In addition to discussing the performance of the system, including submicron positioning accuracy, micron resolution, and fluorescence capability, we demonstrate its relevance for diagnosis in resource-limited settings by imaging several prepared tissue and blood samples, including those containing blood- and fecal-borne human and animal parasites.

## Materials and methods

### Biological samples

Fixed and stained tissue samples (ovary, blood smear, spinal cord, and stratified squamous epithelium) were purchased as part of an educational slide set (SIGA, Suzhou Condor Optical Co., Ltd.). Human parasite samples (*Microfilaria malayi*, *Schistosoma japonica*, *Taenia minima*, *Trypanosoma evansi*, *Trichuris suis*, and *Clonorchis sinesis*) are part of the teaching collection of the Human Parasites Lab of Anhui Medical University, and were graciously loaned for this study. The samples were collected from blood or feces and prepared using standard protocols. Animal parasite samples (*Trypanosoma evansi*, *Eimeria*, and *Toxoplasma gondii*) are teaching samples from the personal collection of Q. Xu, taken as part of routine clinical practice and not for the purpose of this study. These samples, collected from blood and feces, were also prepared using standard protocols[40].

### Optical system

**Fig 1:**
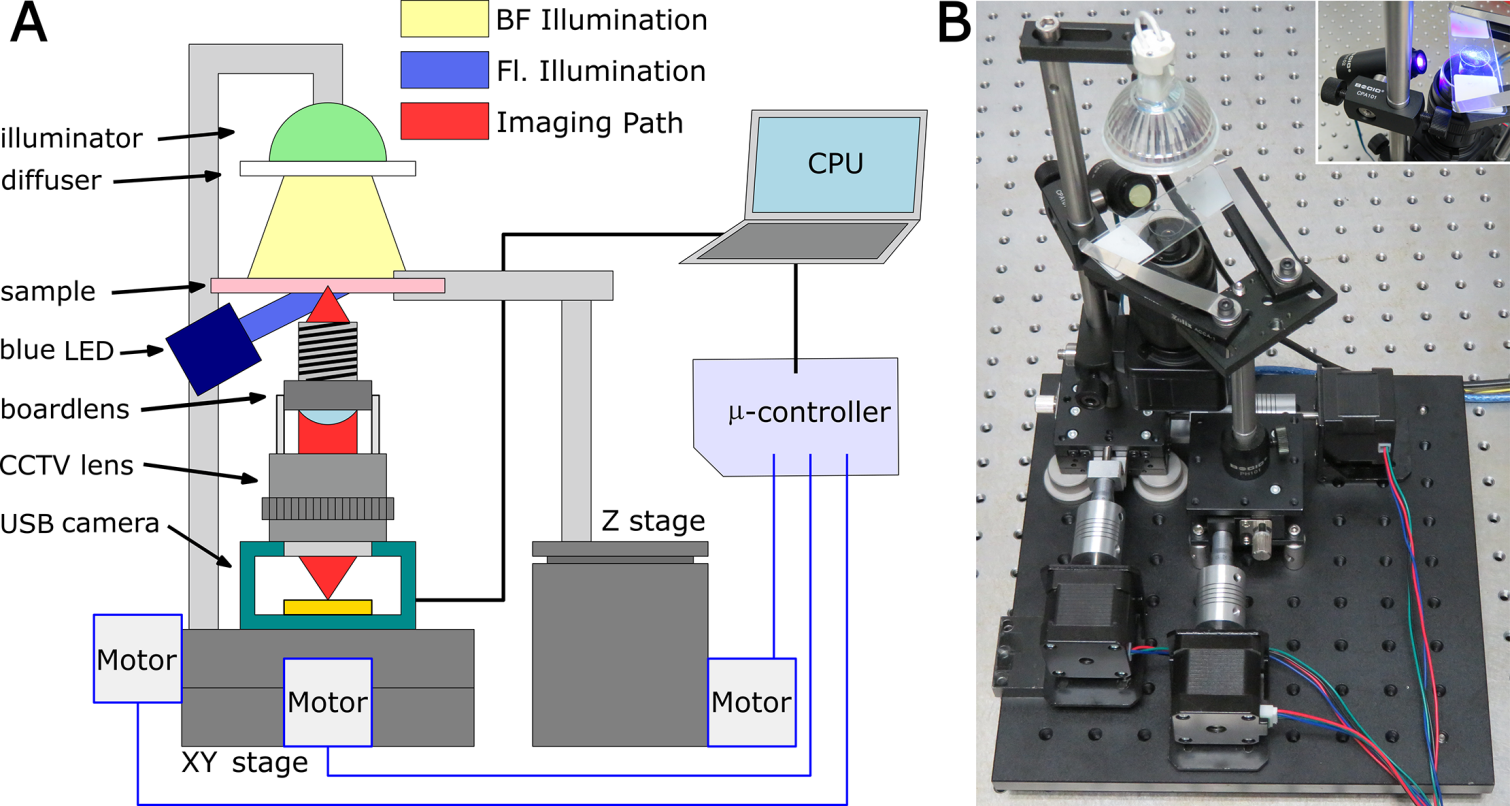
Structure of the low-cost automated microscope. (A) Schematic diagram showing electrical connections and optical path. (B) Photograph of the as-built system.

The structure of the low-cost microscope and stage is shown in Fig 1. The microscope is inverted to allow easy access for sample placement and manipulation. As seen in Fig 1A, the optical system is composed of a simple “4f” optical arrangement, typical of modern infinity-corrected microscopes. A key element of our optical configuration is the replacement of the high quality microscope objective by a low cost “board lens.” Typical microscope objectives are quite costly due to their superior aberration corrections such as flat field compensation and chromatic correction. However, in many cases these higher order corrections are not necessary. As aberrations scale with the numerical aperture and with the field location, a microscope system utilizing modest numerical apertures and with a restricted field of view can still form high quality images even if the objective lens has few surfaces to correct for aberrations. For example, in a previous publication we showed that a simple ball lens can produce high resolution images within a restricted field of view [15]. Board lenses, and other short focal length imaging lenses (such as those used in cell phones), traditionally expect objects to reside at infinity, and form an image approximately one focal length from the lens. Inifinity-corrected objective lenses, by contrast, take an object residing one focal length from the lens and project it to infinity. Thus, a low cost board lens placed in a reversed orientation (i.e. with the object placed where a sensor would typically reside) acts like an objective lens with improved optical performance compared with simpler systems such as ball lenses [41,42]. Our optical system is completed by a CCTV lens (adjusted to be focused at inifinity) acting as the tube lens, and a USB CMOS camera as a detector. The sample is illuminated by a white light LED light bulb that has been covered with diffusive tape, approximating Köhler illumination. The objective lens has a focal length of 4mm and an f-number (f/#) of 1.4 (corresponding to a numerical aperture (NA) of 0.35), designed for a 1/3” sensor. The tube lens is a standard 35 mm focal length CCTV lens (f/# 1.7). As the distance between the two lenses is not critical, we connect them together using a matched bearing housing. The CCTV lens itself has a standard C-mount threads and can be directly connected to the sensor. Details of each piece of the opto-mechanical system, including part numbers, prices, and specifications, are given in S1 Appendix.

### Moving stages

Typical low-cost microscopes, including those commercially designed for classrooms or clinics, or toys that attach to mobile phones, are manually driven and lack the ability to take images in modes other than bright field. In designing this system, we adapt the previously-reported methodology of open-loop stepper motors coupled to precision translation stages to achieve reliable and accurate motion control [39]. An integrated X-Y stage translates the entire optical system (including illuminator), while a Z stage translates the sample for focusing. This not only allows automatic focusing and automated sample positioning, but also enables the system to simply and easily tile high-resolution mosaics of large objects. The total travel range of the system is 13 mm in X and Y, limited by the travel range of the X-Y stage. Compared to OpenStage, a key improvement to our system is the replacement of custom-fabricated flexible couplings by off-the-shelf butt-couplers. However, this brings a slight disadvantage in that with direct motor-stage coupling, as the X-Y stage is driven, one of the motors must move along the stage (due to the physical movement of the stage’s upper axis). To aid in this, the spacer on which the moving motor is mounted has a layer of transparent tape added to reduce the friction between the spacer and the microscope baseplate.

### Autofocusing, auto-scanning, and auto-mosaicking

To reduce the requirements on user training, and enhance the performance of the system for field use, the system was controlled with software that allowed for automated sample positioning, focusing, scanning, and mosaicking. All motion control was performed by a custom-written graphical user interface (GUI) in LabVIEW. Preliminary image analysis was also performed in LabVIEW, but final image mosaicking was performed using Fiji as described below.

To eliminate the need for the system to be focused by hand by a potentially untrained user, the image is auto-focused by maximizing the Brenner criterion [43], a robust image-based focus metric. Typically, the size of a sample is much larger than a single microscope field-of-view. In some situations (for example parasite identification), examining individual fields-of-view is sufficient. However, in other cases, mosaicking multiple fields of view together allows the user to examine the sample morphology at multiple size scales. This can be important for tissue-level pathology or for larger parasites such as flukes. To further reduce the requirements on user training and to enable software-based scanning and tiling of large samples, we also implemented a simple auto-scanning algorithm. The algorithm first acquires an image and then binarizes it to identify if the sample is touching the image boundary in the direction of motion. Thus, for each image, the left margin (LM) and right margin (RM) will be determined to contain the sample or not. Starting at the upper left corner, the algorithm will move right until the RM is clear, then move one row down. If the RM is clear, the algorithm will start to move to the left, and will examine the LM until it is clear before moving down to the next row. If the RM is not clear, the algorithm will first move right and take images until the RM is clear, and then return left checking the LM until the row has been completed. Each time the stage moves and a new image is acquired, the lower boundary (LB) will be checked. If the LB is clear for an entire row, then the auto-scanning program determines that it has reached the bottom of the sample and the scanning will stop at that row.

While this method works for the majority of samples tested, it must be acknowledged that it is a simplistic approach and is not ideal for all samples. It requires that the user position the slide over the camera so that the camera is viewing the upper left corner of the sample prior to starting. Further, if the sample has large holes or a highly irregular shape, the scanning algorithm may miss segments of the sample. For complex objects, asking the user to define an approximate width and height of the sample and performing measurements on a regular rectangular grid (even if that grid contains many blank images), was both simpler and more effective.

To mosaic the images together, we make use of the open source “Stitching” plug-in included as part of the ImageJ distribution Fiji [44]. Image mosaicking programs typically operate by blending the edges of multiple images together, where the mosaicked sub-images intentionally have some small spatial overlap. The physical size of the object seen within one field of view taken by our microscope is about 680 μm x 510 μm. Thus, in our auto-scanning program, the movement between adjacent fields of view is 540 μm in X or 410 μm in Y, corresponding to a 20% overlap. “Stitching” is a powerful plugin with multiple modes of operation. In our implementation, as detailed in S1 Appendix, we utilize the “grid-collection” mode, which expects all mosaics to be rectangular in shape, and the images must be named according to their position within the rectangular grid. Because our auto-scanning approach does not guarantee sequential, grid-acquired images, this can present a challenge. To overcome this challenge, we keep track of the X-Y position of each image acquired, and automatically rename each image after acquisition based on its relative spatial location within the final acquired image set. For corners or edges that lack images, we automatically insert a previously acquired blank image as a “dummy”.

**Fig 2:**
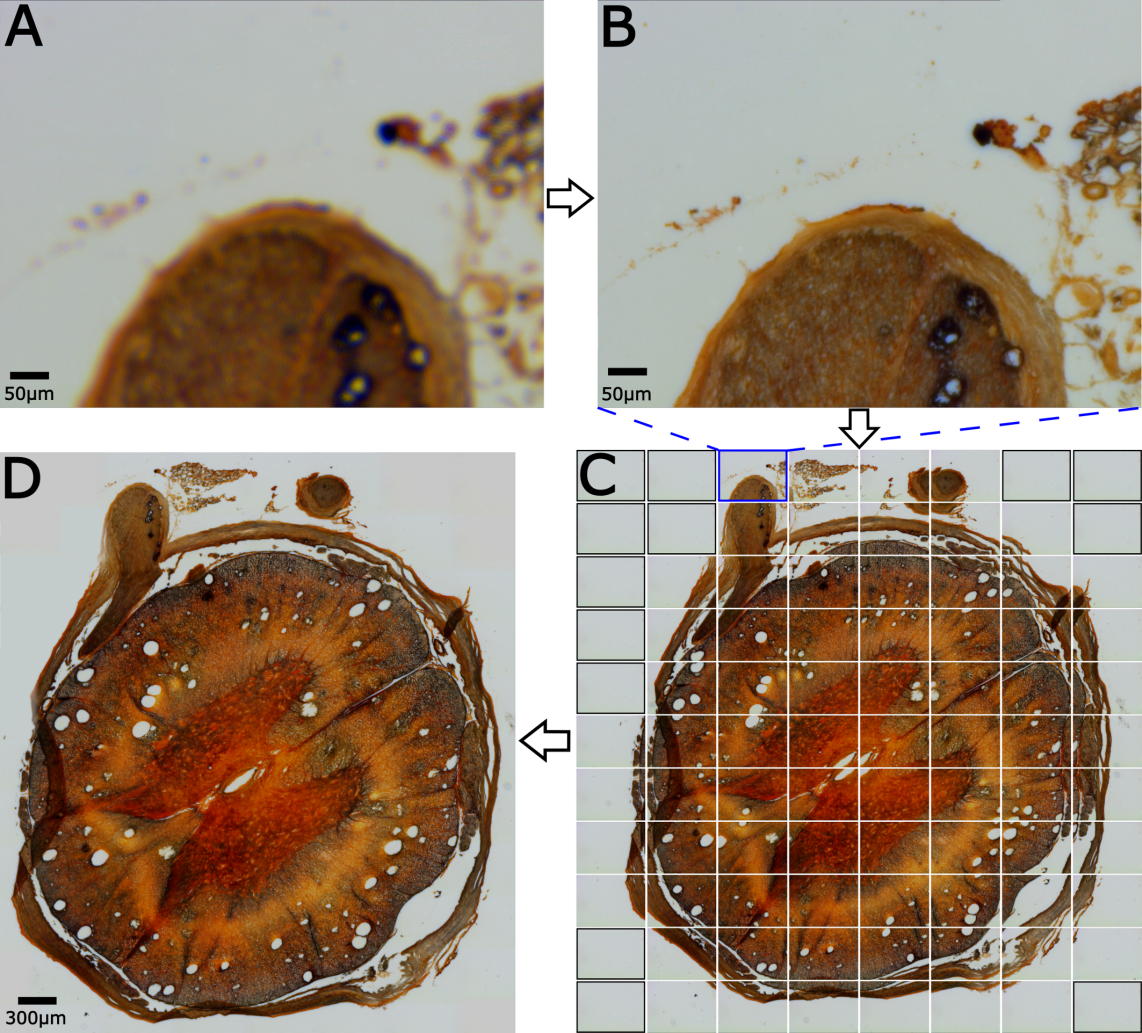
Automated acquisition of large spinal cord section stained with silver staining. (A) User starts at the top left corner. (B) The software first autofocuses the image, then (C) records the individual sub-images. The images with a black outline are “dummy” images automatically added to make the mosaic rectangular. (D) The final mosaicked image.

An example of the complete, automated image acquisition workflow is shown in Fig 2. The first step is to find the top left corner of the sample and start to scan there, as show in Fig 2A. The user can manually rotate the motor shaft or use the directional buttons within the LabVIEW control to perform the initial sample positioning. From this point, the rest of the image acquisition process is fully automated. Fig 2B shows the result of the autofocusing. Fig 2C shows the raw sub-images recorded during the mosaic process, along with the dummy images added to aid the mosaicking. Prior to mosaicking the sample, the individual sub-images are corrected for non-uniformities in the illumination across the field of view. Several blank images are recorded, and from these a median image representing the intensity of the LED source across the field is computed for the red, green, and blue channels of the detector. This image is smoothed with a Gaussian kernel and scaled to have a maximum value of 1. All future images are then divided by this image to flatten the overall intensity distribution. This greatly reduces the tiling artifacts. Once the images have been intensity flattened, and the acquired images have been merged with dummy images to obtain a rectangular grid, the final mosaicked image is shown in Fig 2D.

## Results

### Optical performance

**Fig 3:**
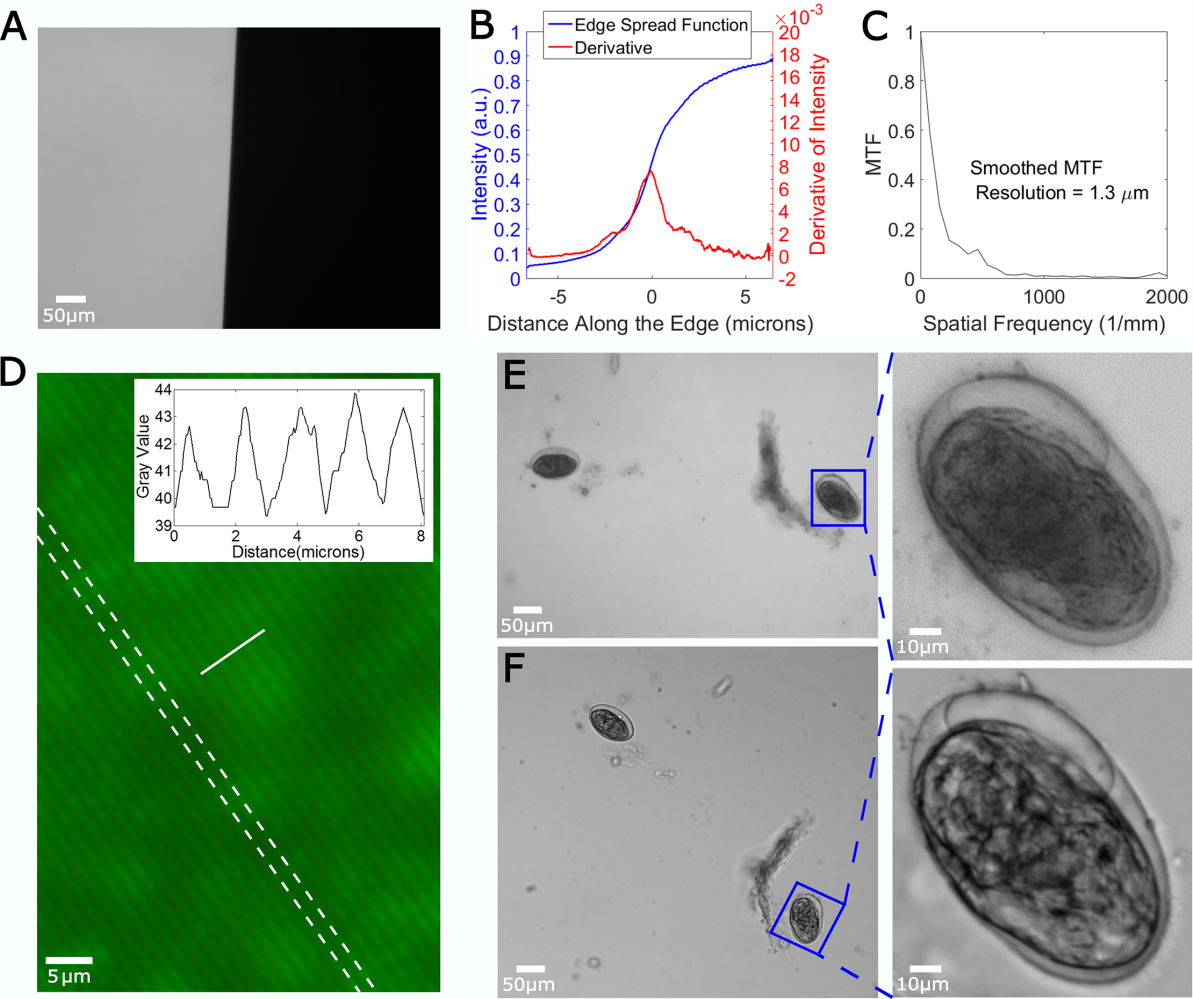
Optical performance of our microscope. (A) Slanted edge image, (B) edge spread function and derivative, (C) estimated modulation transfer function (MTF) showing approximately 1 micron resolution. (D) 600 lp/mm fluorescent Ronchi ruling with diffusive backing, dashed lines show ruling orientation, solid line location of line profile (inset) showing the line pairs are well resolved. (E) Low-cost microscope (NA 0.36) and (F) high-end microscope (NA 0.4) images of fecal smear containing *Schistosoma* eggs. Enlarged ROIs show similar optical qualities.

In order to evaluate the performance of our system, we determined the system resolution based on the classic slanted edge test [45], followed by a qualitative comparison between images taken by our system and images of the same field of view taken by a high-end, commercial microscope (Nikon TIE) Equipped with a high quality objective of similar numerical aperture (20x 0.4 NA). These results are shown in Fig 3. Fig 3A shows the slanted edge imaged by our system (derived from a standard USAF 1951 resolution target). By taking the derivative of the edge spread function (Fig 3B) and Fourier transforming it (Fig 3C), we obtain an estimate of the cutoff frequency of the optical system, defining the resolution limit to be approximately 1.3 microns, in reasonable agreement with the theoretical value of ~0.9 microns (assuming f/# 1.4 and center wavelength 550nm). This can be further confirmed by looking at a 600 lp/mm Ronchi ruling target shown in Fig 3D, where we can clearly resolve the individual line pairs (1.67 μm spacing). A qualitative comparison between the ultra-low-cost microscope and a high-end commercial microscope was also performed using a fecal smear containing *Schistosoma* eggs (Fig 3E and 3F). Despite the markedly different costs of the two systems (representing approximately 100-fold difference in price), the overall field of view, field flatness, and resolution of the two systems are surprisingly similar. A magnified ROI is also presented for detailed comparison. While the high-end microscope seems to have an improved depth of focus and higher contrast (likely due to utilizing true Kohler illumination with a high NA condenser lens), the quality of the reversed board lens is still acceptable.

### Mechanical performance

**Fig 4:**
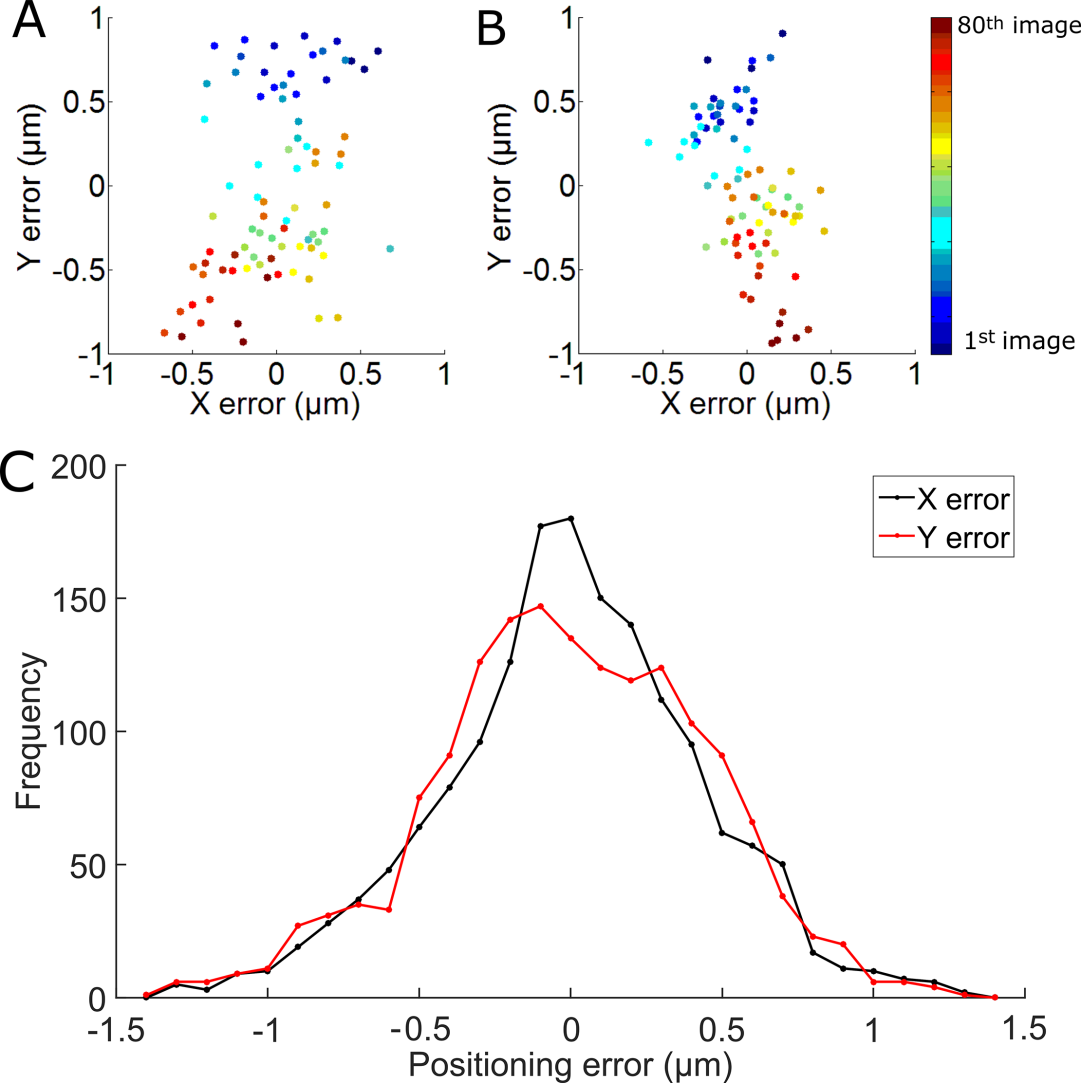
Accuracy of the X-Y stage. Bidirectional repeatability for 80 repetitive motions between two fields of view separated by 950 μm in the X and Y direction. The cloud of datapoints in start position (A) and end position (B). (C) Histogram of errors across 10 independent trials.

To test the mechanical properties of our system the positioning repeatability of our stage was measured using an image-based testing protocol described previously [39]. Briefly, the mechanical system moves repeatedly between two positions A and B, taking a picture of a test object at each location. The bi-directional positioning accuracy can be determined by using consecutive images at each location. Using standard image registration methods, the ΔX, ΔY and ΔΘ between two consecutive images (for example A_1_ and A_2_) can be obtained. Fig 4 shows the X and Y accuracy using this analysis method. In Fig 4, we see the errors due to 80 repetitive motions between point A and point B. The result shown in Fig 4A corresponds to the errors computed from image A, while the result shown in Fig 4B corresponds to the errors computed from image B. In each case we choose the first image in the sequence as the reference image. Because of the cumulative and non-random nature of the errors (due to hysteresis in the mechanical system), the cloud of datapoints may not be centered at (ΔX, ΔY) = (0, 0). To aid visualization, as well as to aid combining multiple datasets together, the cloud is re-centered to (0,0) in Fig 4A and 4B. Points are color-coded to their time, with early points shaded dark blue and late points colored yellow, indicating some clear time-dependent drift in the data. From this image we can see that the errors are less than 1 micron on average. To further validate this, Fig 4C shows the results of combining ten independent trials with a total of 1600 repetitive motions between two points A and B. The majority of points (>76%) are within μ0.5 μm of the origin, while >97% are within ±1 μm of the origin. In addition to the tests shown in Fig 4, we also evaluated the bi-directional accuracy of the stage for the X and Y directions separately, with similar results to the combined XY motion shown above. In this case, more than 99% of the points are within ±1 μm of the origin. As described above, when acquiring image mosaics, each image has about 100 μm overlap on all sides to aid in stitching. Thus, the small errors described here have no impact on the final mosaic, but may be relevant for other uses of the stage such as repeated visits to various locations within a culture dish over time.

### Images of human and animal parasites

**Fig 5:**
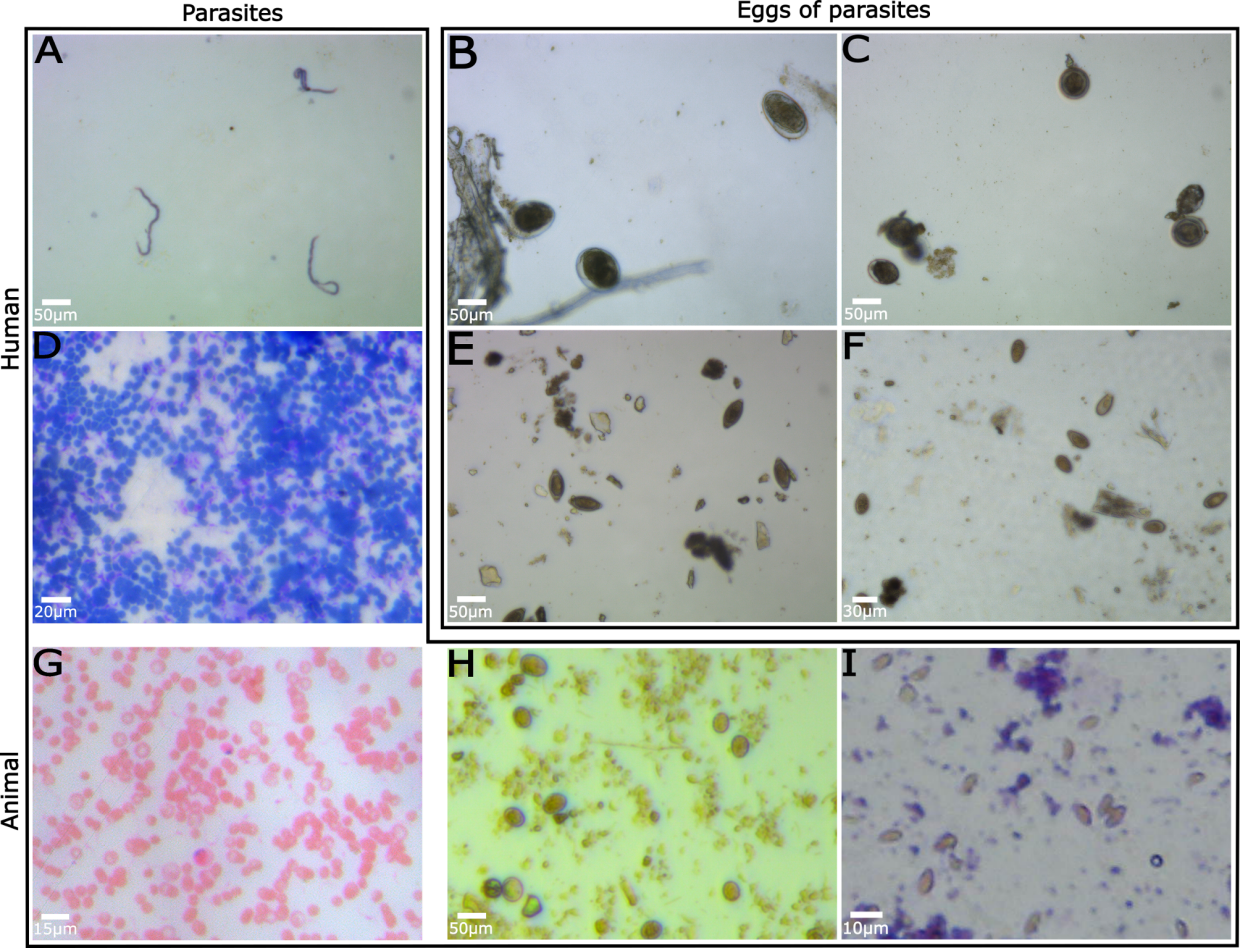
Images of human and animal parasites. (A) *Microfilaria malayi*, Wright-Giemsa stain, (B) *Schistosoma japonica* eggs, (C) *Taenia minima* eggs, (D) *Trypanosoma evansi*, Wright-Giemsa stain, (E) *Trichuris suis* eggs, (F) *Clonorchis sinensis* eggs, (G) *Trypanosoma evansi*, Giemsa stain, (H) *Eimeria oocysts*, (I) *Toxoplasma gondii* trophozites, Giemsa stain.

To demonstrate the ability of the low-cost automated microscope system to effectively aid in disease diagnosis, we used our system to image several fecal and blood smears containing uni- and multi-cellular parasites, as well as parasite eggs, as shown in Fig 5. These common parasites infect both animals and humans, and we include several examples of parasites with veterinary relevance (Fig 5G–5I). Human parasite slides were generously loaned to us by the Laboratory for Human Parasites at Anhui Medical University. As can be clearly seen in these images, the microscopy system, although limited in resolution compared to traditional high NA microscope objectives, still retains the power to accurately diagnose several parasites. For example, *Trypanosoma* and *Toxoplasma* species are characteristically less than 10 microns in length, yet our system retains the ability to unambiguously diagnose these diseases in blood and fecal smears. Note that coloration in the images is due to varying slide preparations, as detailed in the figure captions.

### High resolution images of large samples

**Fig 6:**
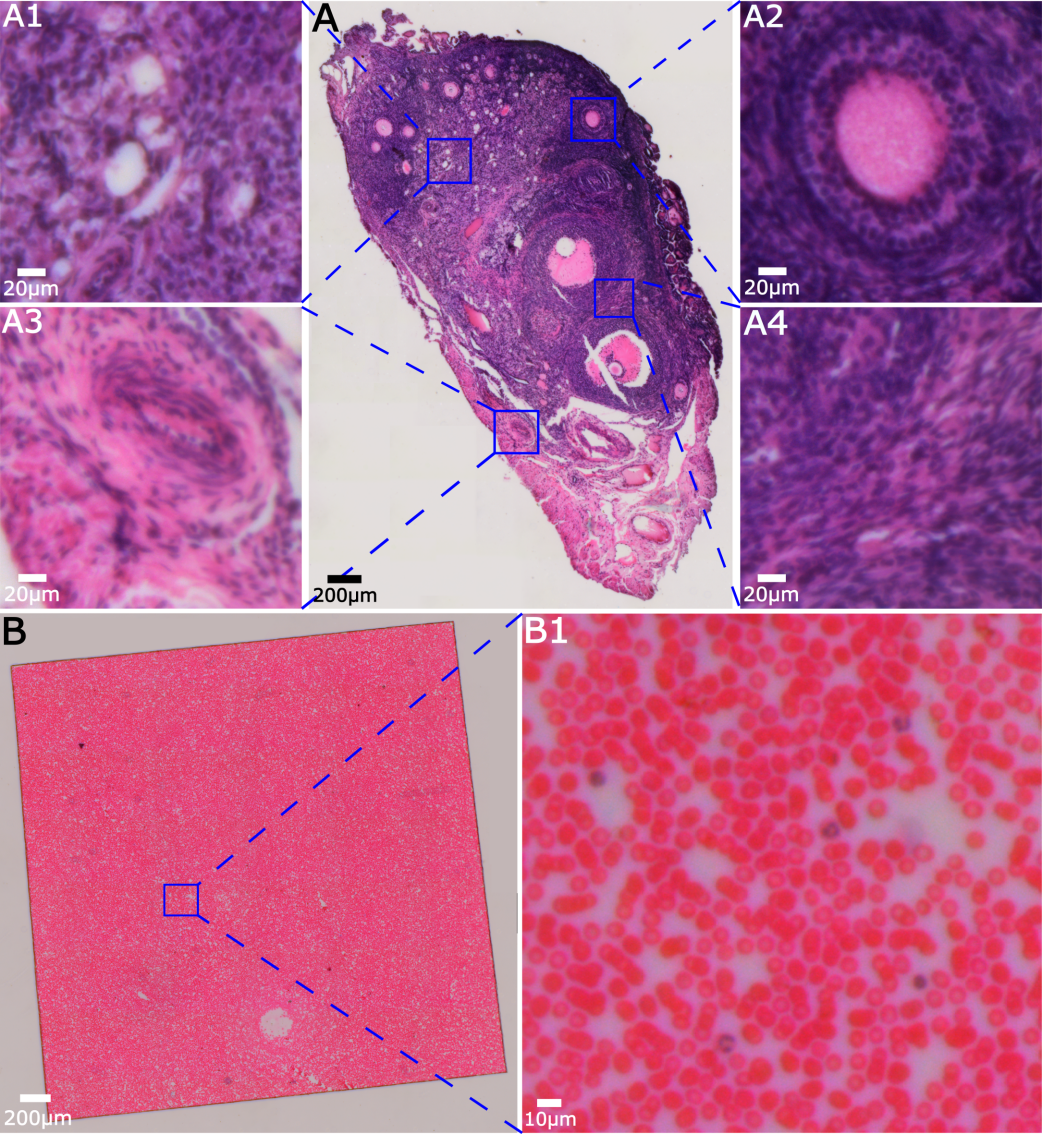
Mosaicked images of (A) an H&E stained mouse ovary section, and (B) a Wright-Giemsa-stained human blood smear.

To explore the utility of the microscope system to automatically image and mosaic samples with relevance to disease diagnosis, Fig 6 shows large-field-of-view images of an ovary (Fig 6A) and a blood smear (Fig 6B). In both cases, enlarged ROIs are shown to demonstrate the ability of the system to image microscopic detail. Note, for example, the whorled structure of the nuclei in primordial follicles (Fig 6A3) compared to the circular, regular nuclei near the oocyte in Fig 6A2. As tissue sections are relatively thick compared to the cell smears presented in Fig 5, the loss of sectioning shown in Fig 3E (compared to Fig 3F) becomes more significant. Adjustments to the condenser system to provide a larger illumination aperture that more closely approximates the Köhler condition may improve this shortcoming. Inspection of the ROI in the blood smear clearly demonstrates the ability to identify the complex, lobed, nuclear structure of granulocytes (lower left, middle, and upper right of ROI) compared to the simple circular structure with little surrounding cytoplasm characteristic of lymphocytes (mid-lower right of ROI). Such images could be used for performing preliminary red and white blood cell counts to help make initial determinations of patient health. With the addition of fluorescence imaging, as discussed in the next section, platelet observation as well as enhanced WBC identification may be possible [46,47].

### Fluorescence imaging

As shown in Fig 1A and the inset of Fig 1B, the first step to add fluorescence functionality to the microscope is to add a low-cost, narrow-cone blue LED (OD469L, Optodiode Inc., center wavelength 470 nm, angular divergence 7 degrees) that illuminates the sample at an extreme oblique angle. The oblique angle prevents the excitation light from entering the microscope objective, and simplifies the task of filtering the excitation from the emission light in the imaging path. A low-cost (and hence basic quality, OD~2 beyond the passband) optical filter with a 15 mm diameter 470±10 nm bandpass filter is placed in front of the LED to limit its emission bandwidth. Both LED and filter fit snugly inside of a standard optical post holder, allowing for easy installation and alignment. The second step is to add an emission filter between the objective and tube lens. In our work we chose a green filter (25 mm diameter, 530±10 nm), allowing us to easily image nuclear fluorescence from acridine orange, as well as the native fluorescence of eosin. The filter was simply placed between the two lenses.

**Fig 7:**
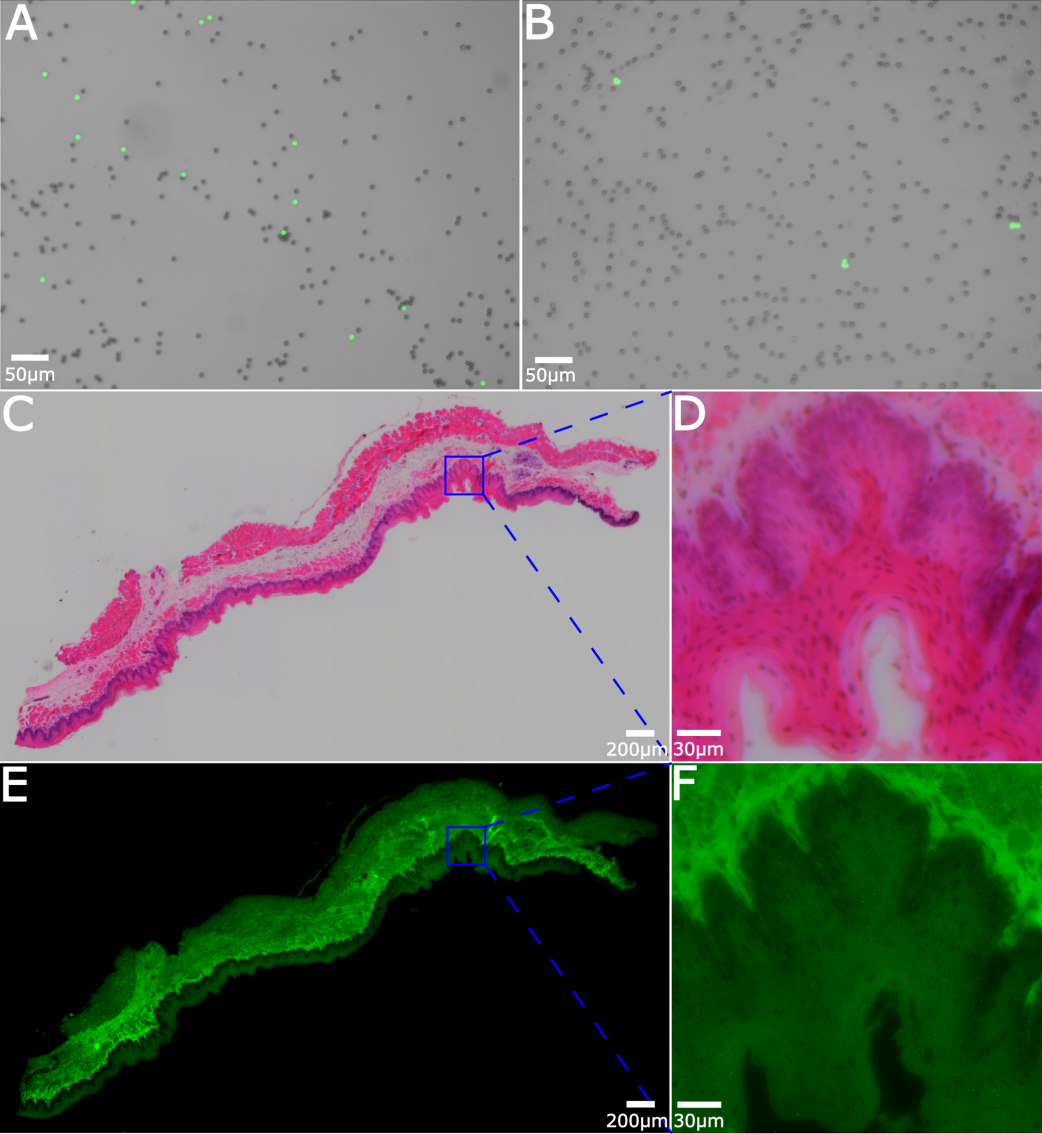
Fluorescence imaging. (A) Merged fluorescent and bright field images of fluorescent and nonfluorescent microspheres. (B) Merged fluorescence and bright field images of blood stained with acridine orange. (C) Bright field mosaic of H&E stained stratified squamous epithelium, with (D) showing an enlarged ROI. (E) A fluorescence image of the same sample as in (C) with (F) showing an enlarged ROI.

Fig 7 shows several representative results of the system operating in fluorescence mode. In Fig 7A, a mixture of fluorescent and non-fluorescent 5 μm polystyrene beads were imaged in both fluorescence and bright-field modes, with the fluorescent image false-colored green and overlayed on the bright field image. Fig 7B shows diluted whole blood stained with 12 μM acridine orange. Red blood cells are visible in the bright-field image, while nuclear fluorescence from white blood cells is clearly seen in the false-colored fluorescence channel. Such an image taken over a large field of view could easily yield red and white blood counts. Combined with CD4 and CD8 antibody staining, the system could potentially be used for HIV screening. Because the fluorescence filter is sandwiched between the two lenses, converting between the two modes requires removing and replacing the board lens. For liquid samples that can move due to Brownian motion, or could be jostled out of position, this presents a limitation in that color images and fluorescence images cannot be simultaneously acquired. In Fig 7A and 7B, therefore, the emission filter was left in the system when acquiring the bright field images. Thus, only the green channel of the RGB image was kept and converted to monochrome.

For fixed samples, it is relatively easy to switch between modes without major disturbances to the sample. Fig 7C and 7E show a large field of view, mosaicked, H&E-stained stratified squamous epithelial tissue sample in bright field and fluorescence modes, respectively. In order to acquire both color bright-field images and fluorescence images, the sample was imaged twice (once with the fluorescence emission filter present, and once with it removed). From Fig 7E we can see that the mosaicking works equally well with fluorescence as with bright field images. Fig 7D shows an enlarged ROI of the crenulated dermal-epidermal junction. As seen clearly in Fig 7F, the collagen-rich dermis is heavily stained with eosin and yields a strong fluorescence signal, while the epidermis has relatively lower fluorescence strength, particularly in regions occupied by nuclei (stained purple in Fig 7D).

## Discussion

In this paper, we report a slide-scanning microscope that is modular, open-source, fully automated, cost-effective, and made of off-the-shelf components. As our targeted use is in healthcare delivery in rural and low-resource settings, as well as combat and disaster areas, high quality images should be acquired with minimal requirements on an expert user. As lack of trained microscopy technicians is a bottleneck to diagnosis in many clinical settings[48,49], the development of automated systems capable of auto-focusing and auto-scanning are sorely needed. To this end, our robotic system has the ability to autofocus, auto-scan, mosaic, and operate in both brightfield as well as fluorescence mode. Its medical relevance has been demonstrated by observing a wide range of parasites and eggs in simply-prepared smears. However, we note that some diagnostic tasks, particularly malaria diagnosis, require resolutions approximately double what is obtained here (typically NA~0.8 or higher). Therefore, our system is clearly not appropriate for all diagnostic tasks. Nevertheless, it remains highly relevant for several key applications such as water quality assessment, fecal parasite detection and counting, *in situ* cell culture or fungal culture monitoring, among others. The high precision of our system is not strictly required for X-Y scanning of a flat samples, however high Z precision allows robust autofocusing for imaging non-flat samples (or simply overcoming a tilted Z-stage) and high X-Y precision can be used to automate periodic monitoring of culture dishes or other longitudinal experiments.

We also note here that our system has applications in resource-constrained settings beyond healthcare. For example, in the context of science education, our system offers interested students the opportunity to learn the basics of imaging, how to build and control robotic movement systems, utilize a series of automated algorithms, and provides an introduction to basic biology. Further, if the microscope is considered as a student project, when the project is complete the system can remain in the school as a lasting resource for biology classrooms or other areas of science education. Despite its broad potential use, further improvements can still be envisioned.

Compared with other low cost systems in the literature, our system is the only automated system to rely only on off-the-shelf components. The mechanical structure of our system required careful selection of components to ensure that each part fit together stably, without need for any custom-constructed part or housing. For example, to eliminate the need for custom couplers between the motors and stages, each motor needed a spacer to raise the spindle to the appropriate height to couple to the stage micrometer. Our microscope has a fixed magnification of 8.5, which balances the achievable resolution with the field of view spanned by the sensor. Given that each pixel is about 0.26 μm, we have approximately 4x4 pixels per optical resolution element, placing us comfortably within the Nyquist limit without limiting the field of view. Our resolution is limited by the available f-numbers of low-cost lenses. However, our system is synergistic with advanced imaging techniques such as Fourier ptychography, which could be used in the future to improve the achieved resolution by a factor of 2–4. Further, we would be remiss to not acknowledge that our instrument control is implemented currently in LabVIEW, and, while we have made the VIs and a stand-alone binary freely available (as detailed in S1 Appendix), a truly open-source alternative is planned for the future.

Despite the minor limitations of the system as described above, its performance is in other respects on par with that of conventional high quality microscopes operating at modest numerical apertures up to 0.3, with the added benefit of being compact, portable, with automated sample focusing and sample motion. Despite these advantages, the system costs less than US$400 with an additional $100 for added fluorescence capabilities. Therefore we hope that this system will find significant use in healthcare in low-resource settings as well as in educational settings worldwide.

## Supporting information

S1 Appendix Detailed description of each component, along with a stepwise illustration of the construction process, wiring schemes, and software control. (PDF)
