# Supplementary material for: A modular, open-source, slide-scanning microscope for diagnostic applications in resource-constrained settings
Source: PLoS One. 2018 Mar 15;13(3):e0194063. doi: 10.1371/journal.pone.0194063 (PMC5854341; doi:10.1371/journal.pone.0194063)
Supplement: S1 Appendix — (PDF) [file pone.0194063.s001.pdf]

Supplemental Material for "A modular, open-source, slide-scanning microscope for diagnostic applications in resource-constrained settings"

Qiang Lu[1], Guanghui Liu[1], Chuanli Xiao[1], Chuanzhen Hu[1], Shiwu Zhang[1], Ronald X. Xu[1], Kaiqin Chu[1], Qianming Xu[2,*], and Zachary J. Smith[1,*]

[1]University of Science and Technology of China, Department of Precision Machinery and Precision Instrumentation, Hefei, Anhui, China

[2]Anhui Agricultural University, Hefei, Anhui, China

*Address correspondence to: QX: xuqianming2006@163.com, ZJS: zsmith@ustc.edu.cn

Contents

1. Component Details 1
2. Stepwise illustration of the building procedure 3
3. Illustration of wiring scheme 10
4. Program link and brief manual..10
    4.1. Acquiring images and microscope operation. 11
    4.2. Grid scanning 12
    4.3. Image mosaic by Fiji...13
    4.4. Software for USB camera...15

1. Component Details

	Number	Items	Manufacturer	Part Number	Main parameters	Amount	Price/RMB
Optical system	**1**	**Illumination**	Numegv led co., Ltd	MR16	36 LEDs; 7 W; 220V Input	1	17
	**2**	**Objective**	Fujian FuGuang Digital Technology Co., Ltd	CCTV	f=4mm; F1.2; 1/3"	1	10
	**3**	**Tube lens**	Fuzhou Forsete Optical Instrument Co., Ltd	FSLM3514	f=35mm; F1.7; CS connector	1	90
	**4**	**Camera**	Shenzhen Mingshi Wei Technology Co., Ltd	VK-U500	Color camera; CMOS; 2592 x 1944 pixel	1	628
	**5**	**Bearing housing**	Company of YunDing Bearing of Shandong	28-32-20	ID=28mm; OD=32mm; H=20mm	1	4
Mechanical structure	**6**	**Breadboard**	Shenzhen Hengyu Laser Instrument Co., Ltd.	250 x 250	250 x 250 x 13mm	1	310
	**7**	**X-Y stage**	Dongguan Jutian Li Pneumatic Technology Co., Ltd	LY60-CM	Range= ±6.5mm; Minimum scale, 10 μm	1	420
	**8**	**Z stage**	Dongguan Jutian Li Pneumatic Technology Co., Ltd	LZ60	Range= 10mm; Minimum scale, 10 μm	1	200
	**9**	**Stepper motors**	Wenzhou Pufei De	42BYG39	Maximum torque,	3	96

			Electric Co., Ltd.		0.4 Nm		
	**10**	**Motor controllers**	Wenzhou Pufei De Electric Co., Ltd.	DM3230	Maximum 128 subdivision	3	120
	**11**	**Microcontroller**	Arduino	Uno	MEGA 2560	1	53
	**12**	**Optical posts & Holders**	Beijing Optical Century Instrument Co., Ltd	SPF101,	L=25mm	7	105
	**13**			SP124,	L=100mm	2	70
	**14**			SP135,	L=150mm	1	50
	**15**			PH101	L=25mm	1	25
	**16**			PH102	L=50mm	1	35
	**17**	**Mounting plates**	Beijing Optical Century Instrument Co., Ltd	BC205		1	40
	**18**			BP111		1	35
	**19**			BP114		1	40
	**20**			BP118A		1	35
	**21**		Zolix Instrument Co., Ltd	PPB15	H=15mm	4	40
	**22**			ACCA-1		2	40
	**23**	**90-degree post couplers**	Beijing Optical Century Instrument Co., Ltd	CPA101		2	98
	**24**	**Motor-Stage couplers**	Company of Tongjiu Transmission	5-13	Switching diameter: 5-13mm	3	60
**Fluorescence**	**25**	**Blue LED**	Optodiode, Inc.	OD-469L	CWL=470nm	1	209
	**26**	**Excitation Filter**	Shanghai Huoda Xingguang Co., Ltd	M47	CWL=470nm; FWHM=10nm; Diameter=15mm	1	100
	**27**	**Emission Filter**	Shanghai Huoda Xingguang Co., Ltd	M53	CWL=530nm; FWHM=10nm; Diameter=25mm	1	280
**Others**	**28**	**Rubber dampers**	Shanghai Jingfeng Xiangsu Hardware Co., Ltd	D20*H20 M6	M6 screw	4	16
	**29**	**Headless screws**	Thorlabs	SS6MS20	M6 x 20mm	5	8
	**30**	**Cap screws**	Zhongshan Taiming Hardware Co., Ltd	M5-35	M5 x 35mm	4	4
	**31**	**Cap screws & square nuts**	Thorlabs	SH4MS10	M4 x 10mm	2	3
	**32**	**Cap screws**	Thorlabs	SH4MS25	M4 x 25mm	2	3
	**33**	**Washers**	Thorlabs	W25S050	M6	8	4
	**34**	**Microscope slide clamps**	Beijing Tektronixhui Technology Co., Ltd	8.2	L=8.2cm	2	14
	**35**	**Cap screws**	Thorlabs	SH6MS12	M6 x 12mm	1	1
	**36**	**Instant glue**	Shanghai Huancai Industry Co., Ltd	502	Instant glue	1	2
	**37**	**LED mount**	Xingguang Co., Ltd	HS255-ND	Heatsink TO-5 2W black	1	75
	**38**	**Headless screws**	Thorlabs	SS4MS12	M4 x 12m m	2	2
	**39**	**Switching power supply**	Hong Kong Mingwei Power Co., Ltd.	S-50-12	Output: 12 V	1	36

	**40**	**Scotch tape**	Kunshan Weida Si Electronics Co., Ltd	3M600	12mm x 32m	1	2
**Total Cost (RMB / $)**	Bright field only						￥2645/$390
	Plus fluorescence						￥3413/$498

S1 Table. Component list including supplier and source (hyperlink). For those who can't access linked sources online, local equivalents can be easily substituted.

A complete and comprehensive list of components used in the construction of the low-cost automated microscopy system is supplied in S1 Table, above. The list provides a description, part number, and a link to the supplier. As the majority of these supplies are standard opt mechanics or electronics, for those in parts of the world where listed suppliers are not available, equivalent products can be easily substituted. As all purchases were made using RMB, the current exchange rate of 6.7868 RMB per dollar was used when calculating the dollar cost.

2. Stepwise illustration of the building procedure

Where we start.

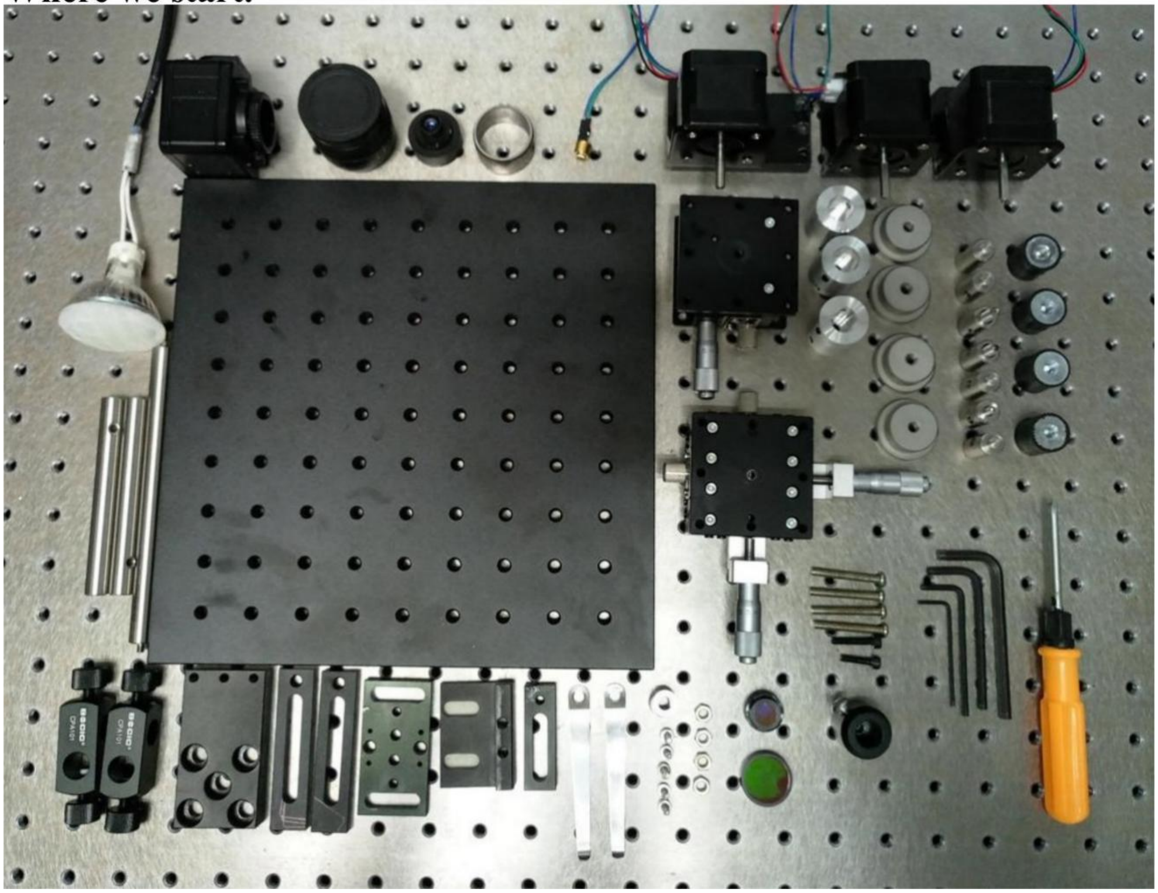

S1 Fig. The picture above shows nearly all the components that will be used to build the low-cost and open-source microscope. This work will be divided into 6 steps, as shown in the following 6 figures. Each component has a given number in S1 Table, and this number will be shown next to the component's position in the following figures. Brief explanations below each figure present additional information and tips for construction.

The 1st step: preparing the baseplate.

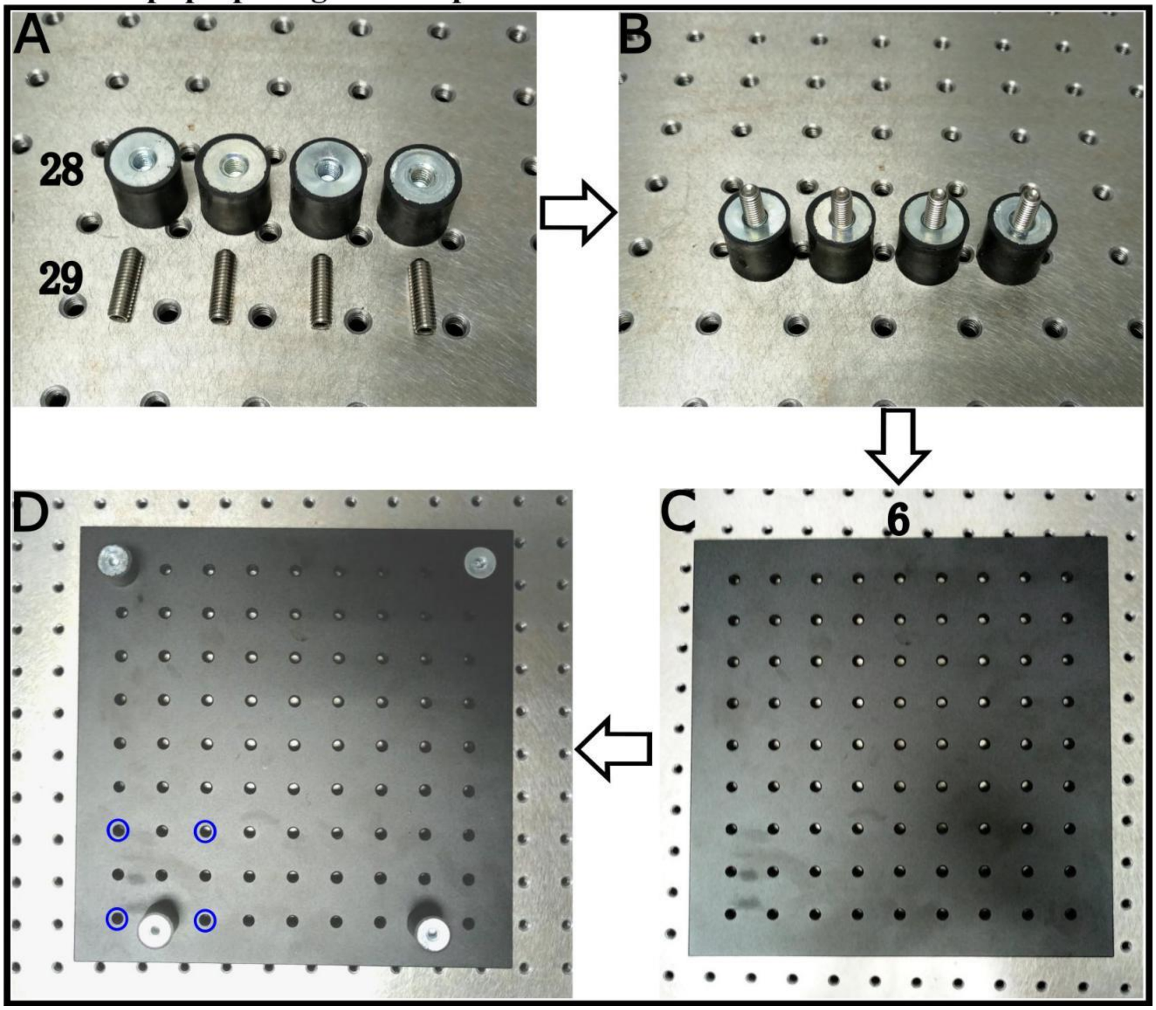

S2 Fig. Both '28' and '6' are tapped for M6 screws ('29'). In (D), the two rubber dampers in lower positions aren't fixed on the corners holes because the screw holes indicated by blue circles will be used to fix the X-Y stage as shown in S3 Fig, below.

The 2nd step: installing X-Y stage on the baseplate.

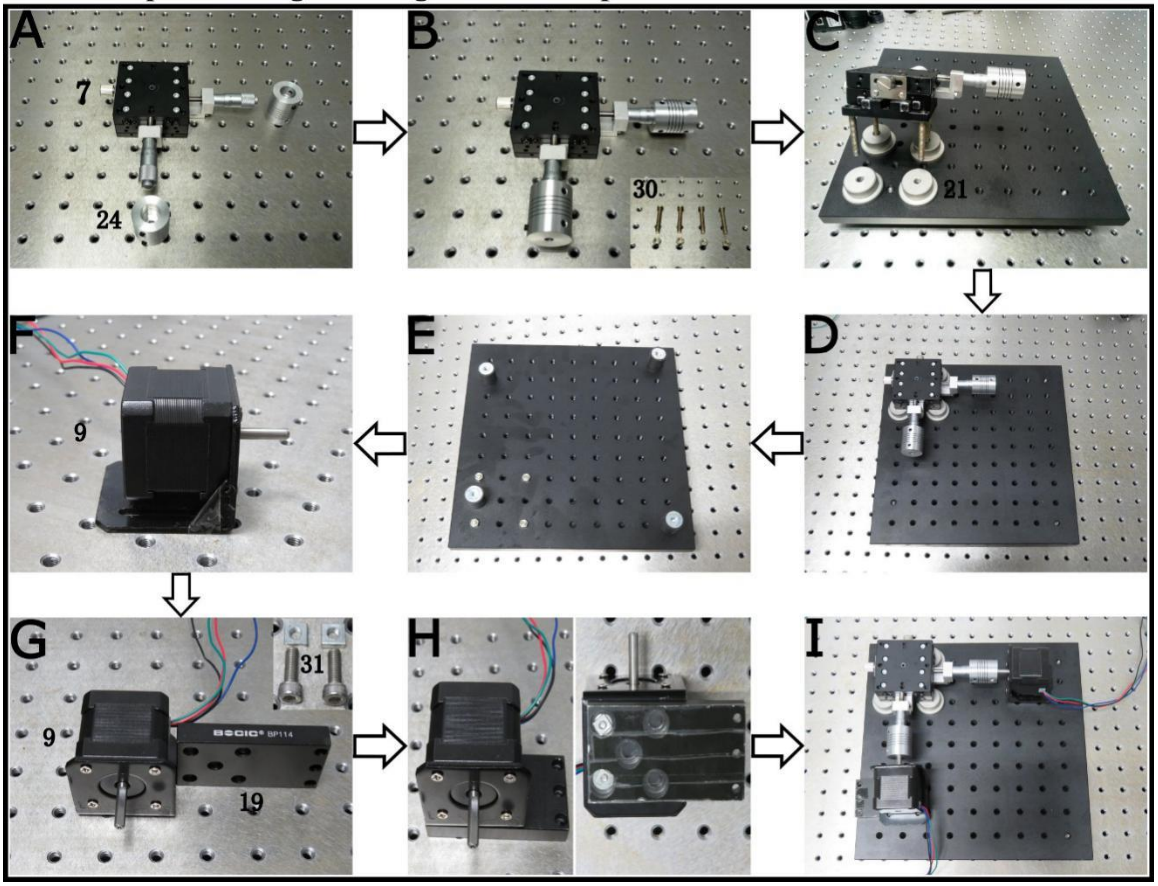

S3 Fig. In (B) and (C), four long bolts ('30' in S1 Table) fit in the four countersinks in the corners of the X-Y stage and screw into 4 nuts to fix them to the baseplate (shown in (E)). '21' (H=15mm) brings the stage to a suitable height for motor attachment. In Figure (G) and (H), the mounting plate ('19') connects with the motor by two sets of short screws and square nuts ('31') to adjust the motor's height. Tape applied to the lower surface of mounting plate (as shown in (H)) helps the motor to move more smoothly on the baseplate.

The 3rd step: installing Z stage on the baseplate.

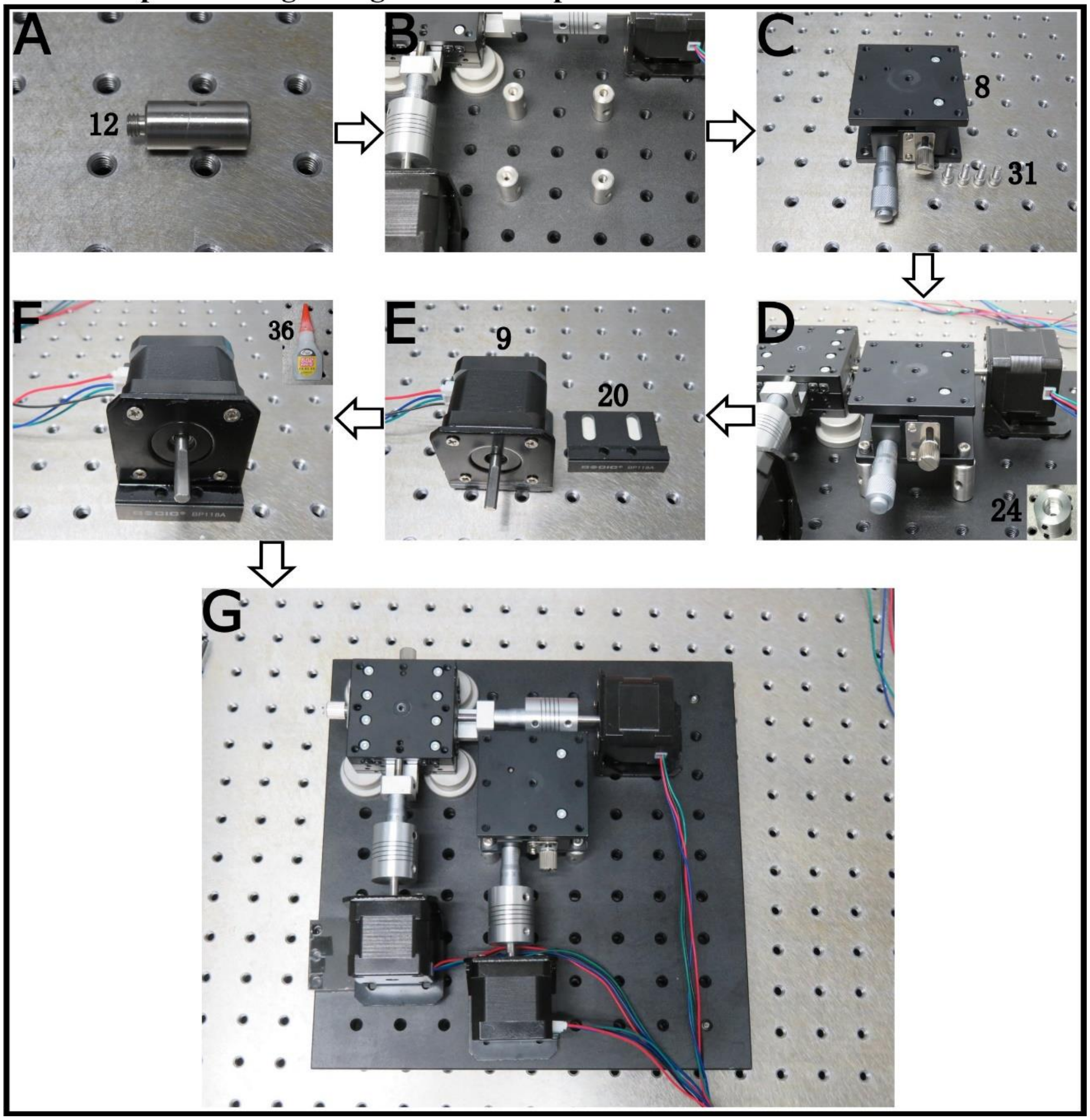

S4 Fig. In (A), '12' has M6 male screw at one end and M4 internal screw at the other end. Four of '12' are fixed on the baseplate using the M6 screw, as shown in (B). Four M4 screws ('31') mount the Z stage to the posts. In (E) and (F), mounting plate ('20') connects with the motor using instant glue ('36'). Only a small bead of glue is required, and the connection is quite stable, yet the two parts can be separated if required by delivering a sharp blow to the baseplate with a hammer.

The 4th step: building the optical system.

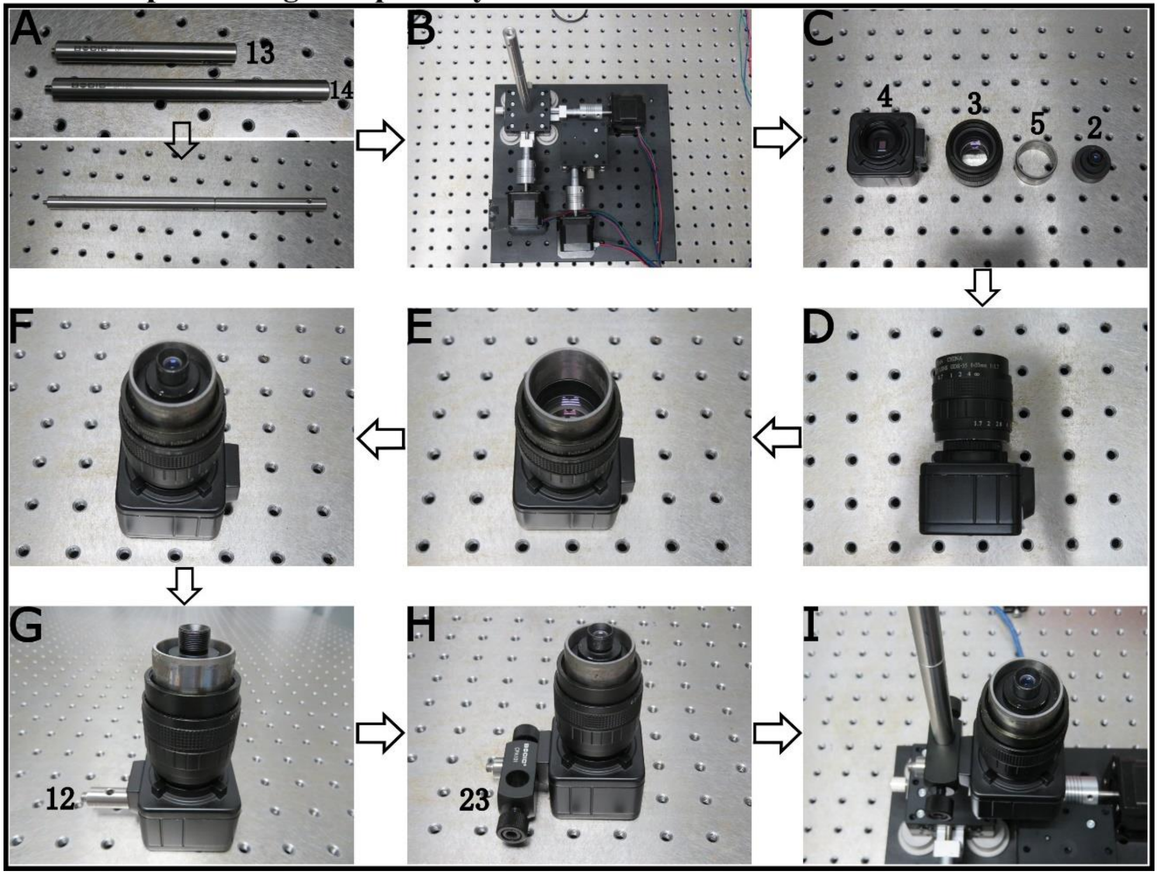

S5 Fig. (A) '13' and '14' can be replaced by a single post 250mm long if desired. (B) The support is erected in the center of the X-Y stage's upper surface through the M4 screw. In (E) and (F), the bearing fits within the CCTV lens's housing, and the board lens fits into the bearing's housing. When fluorescent images are taken, one emission filter will be needed to be placed between the CCTV lens and board lens. In this case, the order of construction in (E) and (F) should be: camera, tube lens, filter, and objective. (I) A 90-degree USB cable may help to reduce cable strain and allow the camera to be placed closer to the table surface.

The 5th step: installing the sample stage.

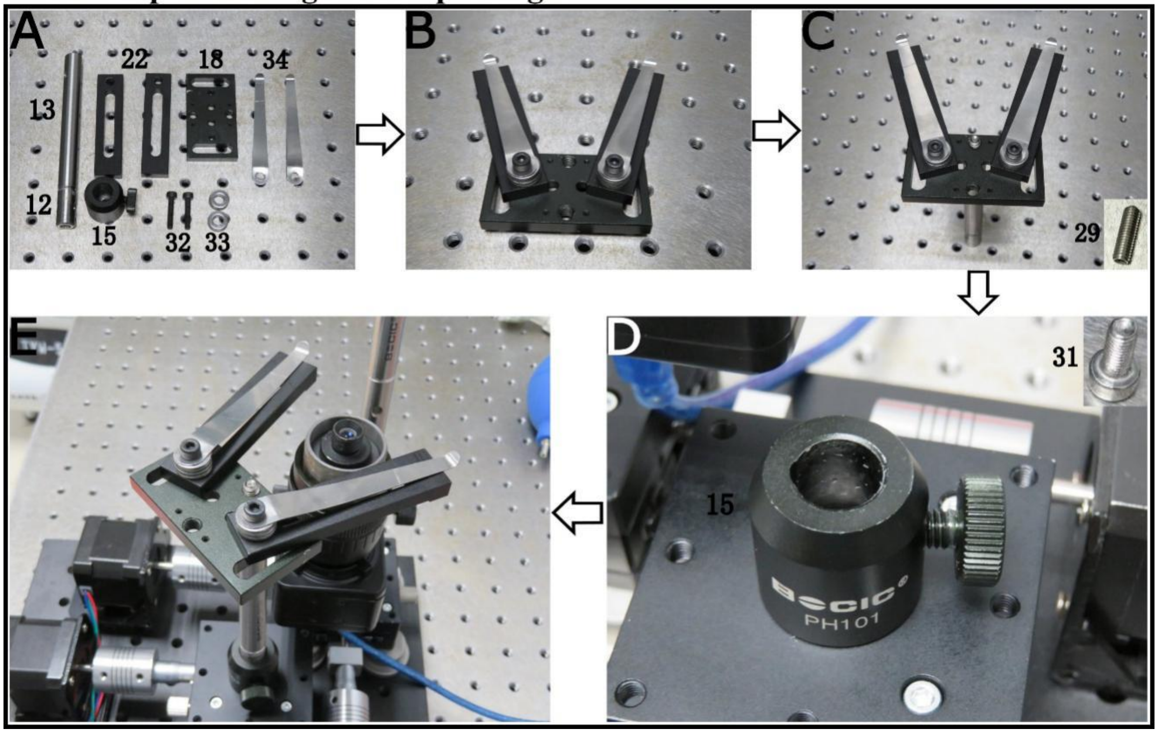

S6 Fig. (A) '12' and '13' can be replaced by one 125mm long post. (B) 4 washers ('33') are stacked for each finger of the sample mounting clamp.

The 6th step: installing the bright field and fluorescence illumination.

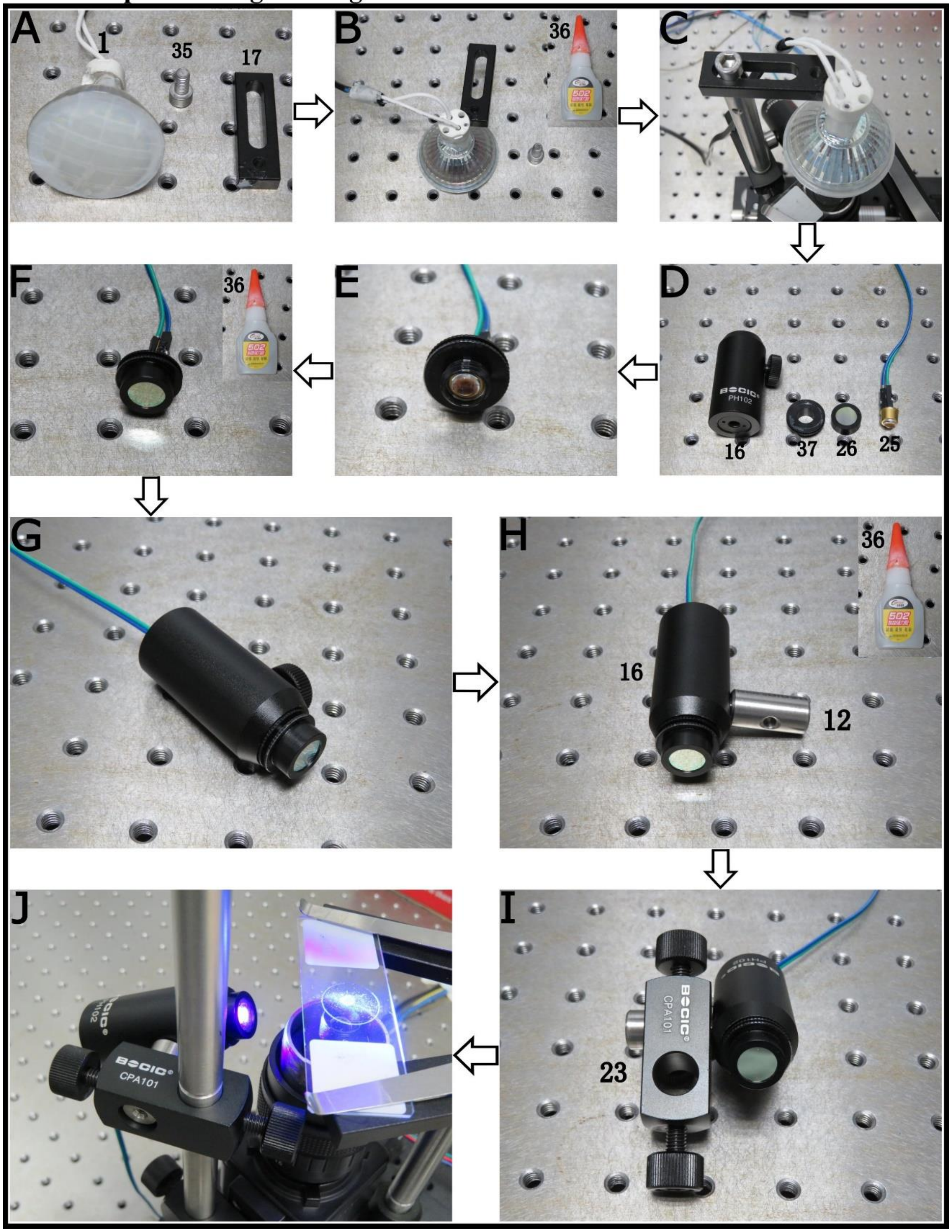

S7 Fig. (A) The LED light bulb is covered with diffusing tape to decrease the coherence of the source at the sample plane and improve the image quality. (B) The mounting plate and illuminator are fixed using instant glue. (E) The LED is matched to a heatsink. (F) The LED is mated with the excitation filter by instant glue at the edges, and the LED-filter assembly is glued to the optical post holder ('16') for mounting.

3. Illustration of wiring scheme

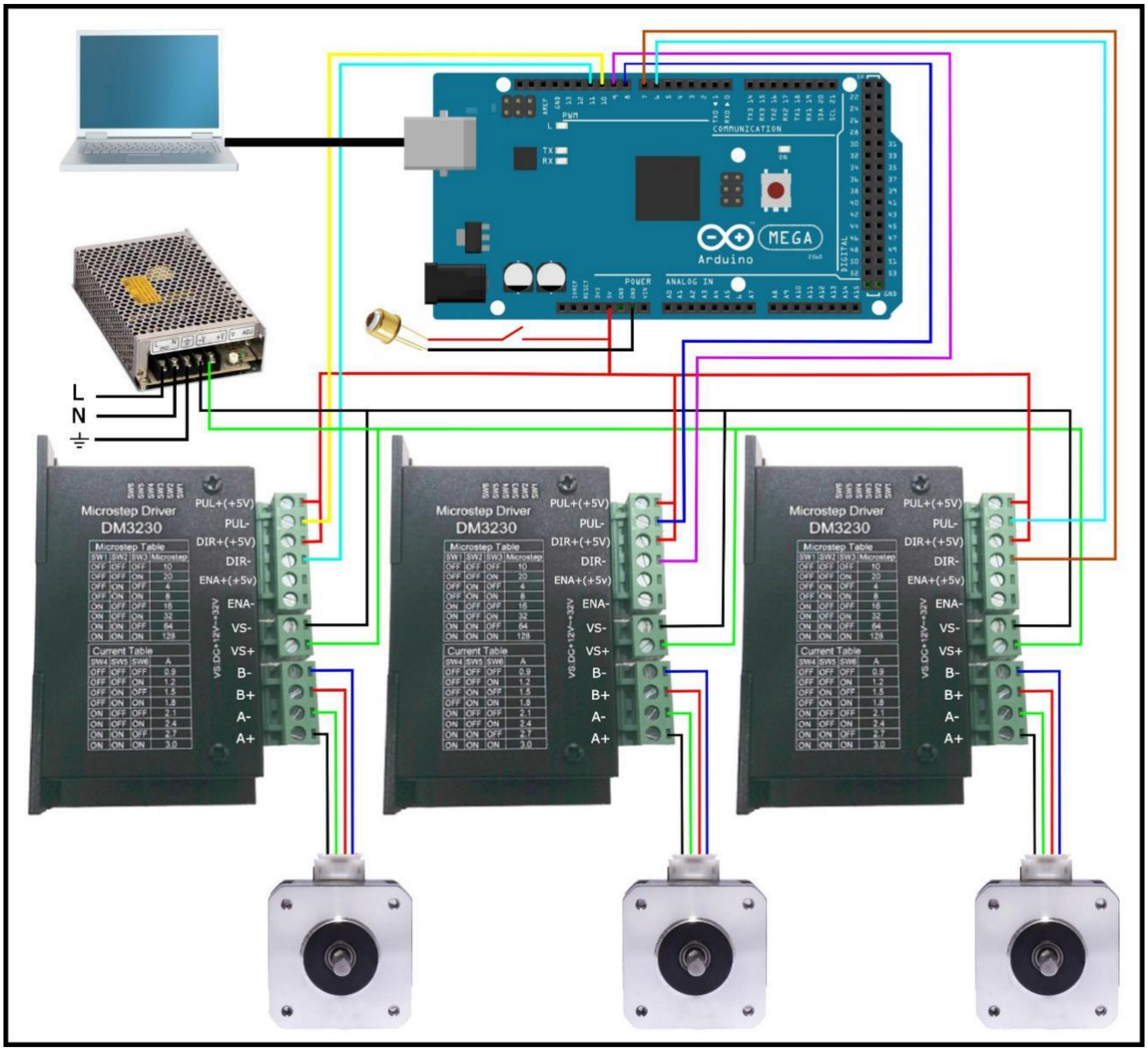

S8 Fig. Wiring and control scheme. Wire colors are used to aid visualization. The colors of the four lines from three stepper motors correspond with the real wire colors. A physical or electronic switch in the LED's circuit can be used to control the blue fluorescent light and shift freely between the bright field and fluorescence modes.

4. Program link and brief manual

A RAR archive containing standalone executable, LabVIEW VIs, etc. can be found at the Figshare repository, DOI: 10.6084/m9.figshare.5931709, or alternatively at http://staff.ustc.edu.cn/~zsmith/research. The package includes four folders and several files, as introduced below.

The computer control of the motors is mediated by an Arduino microcontroller. To provide instructions to the microcontroller, it is necessary to load the Arduino sketch “motor-control” (located inside the folder “Arduino program”) using the Arduino IDE, available at http://www.arduino.org/downloads.

The control software consists of three LabVIEW VIs, “Microscope,” “Microscope-fluorescence,” and “Square scanning,” which we provide so that they can be freely edited by users. For those without access to LabVIEW, these VIs have each also been wrapped into standalone executables, which can be installed by running the “setup” file in the folder “Microscope programs.”

4.1. Acquiring images and microscope operation.

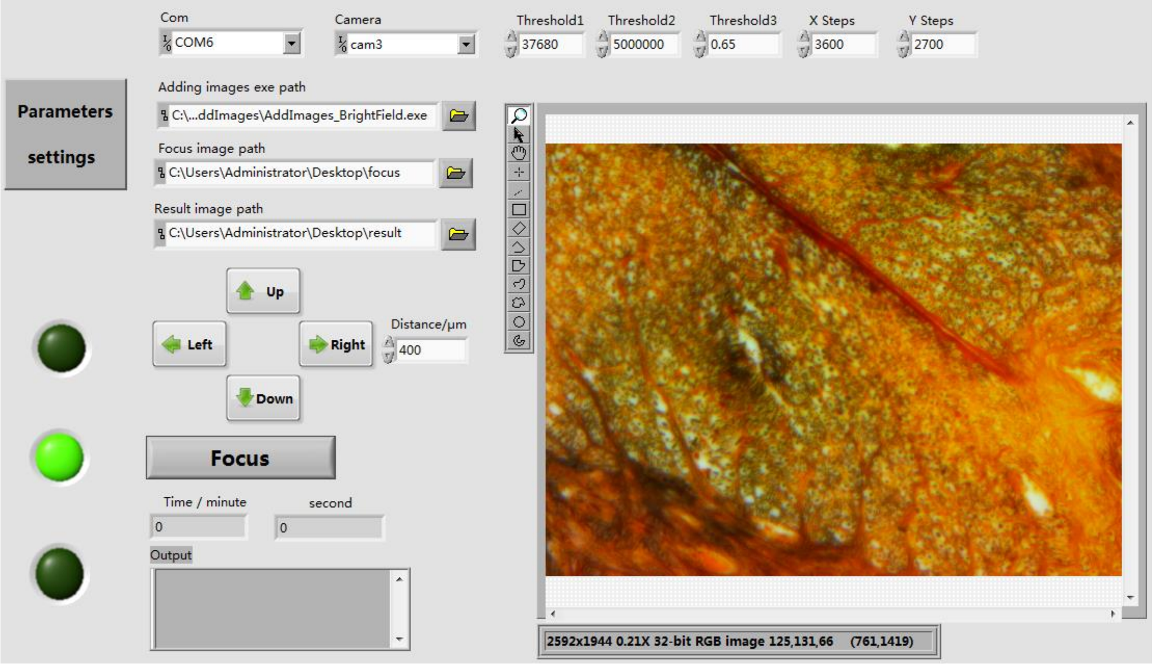

S9 Fig. Control Panel of main program in bright field

The first executable file is ‘Microscope.exe’ which is the main program used for bright field microscopy. The main panel of this program is shown in S9 Fig. The first part of this panel is ‘Parameters settings’. The two options of ‘Com’ and ‘Camera’ are the com port and camera id of the Arduino and USB camera, respectively. ‘Threshold1’, ‘Threshold2’, and ‘Threshold3’ are three threshold values to make judgements in the auto-scanning program and it is recommended to keep the default values. ‘X step’ and ‘Y step’ represent the number of pulses given to the motors between images in the scanning mode. In our system, given the gear ratio of the stages, 5.89 pulses moves the system by 1 micron.

‘Adding images exe path’ should be directed to the ‘Add_images1.exe’ program. This is a MATLAB standalone executable that automatically converts the auto-pathfinding images into a square grid by adding “dummy” images, as described in the main text. It is contained in the “4.Add images exe” folder. Within this folder, the user must click on “Installer” to install the necessary runtime environment for the executable. Once the runtime environment has been installed, set the “Adding images exe path” in the control panel to the exe itself (i.e. do not only provide the folder). ‘Focus images path’ is used to store temporary images used in the auto-focusing, while ‘Result images path’ should be directed to the folder where you want to save the final scanning images.

In the second portion of the GUI are the buttons to operate the program. In the top portion, four buttons allow the user to move the sample in X and Y in a stepwise fashion, moving a distance equal to the number inputted into the “Distance” box. In the middle portion of this section, the ‘Focus’ button will auto-focus the image and also automatically start the auto-pathfinding program. When scanning is completed, the bottom portion of this section will display the total image acquisition time, along with Grid size (x and y) and first file index (x and y). The grid size parameters indicate the column and row size of the full image matrix, while the latter two parameters represent smallest numbers of x and y in the naming system. These four parameters should be noted, as they will be used when running the image mosaic program.

We note here that the second executable file, “Microscope-fluorescence.exe,” which is used for fluorescence imaging, is largely similar to this program. However, the “Adding images exe path” should be set to “Add_images2.exe.

4.2. Grid scanning

While our auto-pathfinding algorithm works for most situations, in some cases users may want to simply scan a grid-sized region of a predefined size. In this case, the user can open the third program, “Square scanning.exe,” whose control panel is shown in S10 Fig. The inputs Com, Camera, X Steps and Y Steps have the same meaning with the corresponding options in “Microscope” programs. “Image path” contains the folder path where result images are saved. “X times” and “Y times” represent the final size of the scanned grid in X and Y. Scanning starts from top left corner of the image matrix and goes right, then down, then left, then down, and so on in a snakelike track. Acquired images are saved with incrementally increasing filenames (0.bmp, 1.bmp, 2.bmp……).

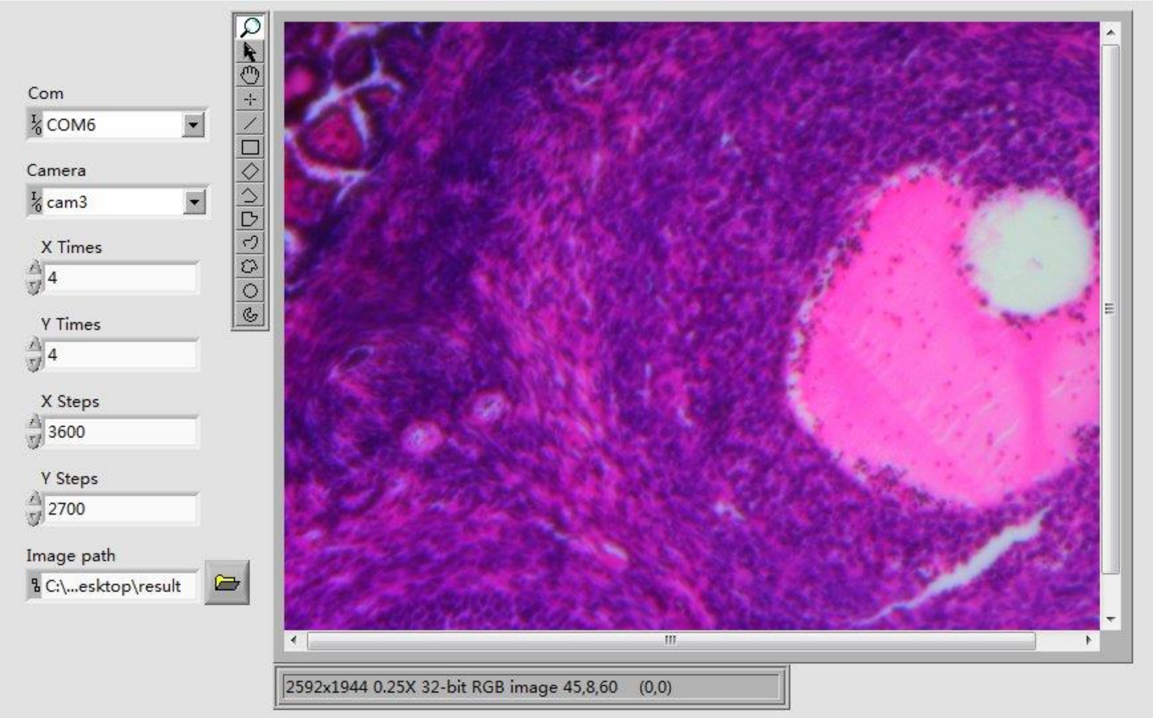

S10 Fig. Control Panel of Square scanning program

4.3. Image mosaic by Fiji

Fiji is a free distribution of ImageJ containing several extra packages compared to the “vanilla” ImageJ distribution, and is available at: http://fiji.sc/#download

To mosaic images acquired by the system, navigate to Plugins->Stitching. In “Stitching,” choose “Grid/Collection stitching” option. S11 Fig lays out the important options to select during this process. Part A describes the case where the auto-pathfinding algorithm has been utilized. In the “Type” option, we choose “Filename defined position.” All the options arrows with red arrows must be filled using the values Grid size x, Grid size y, First file index x, and First file index y from the output of the “Microscope” GUI. The “Directory” option should be set to the location of the image files. “File name for tiles” should be input as shown (-{x}-{y}.bmp). Finally, click the “Ok” button to run the program and the fused image will be shown after several seconds.

If the user collected data using the “Square scanning” GUI, Part B is used for the mosaic process. In the “Type” option, we choose “Grid: snake by rows” and in the lower “Order”

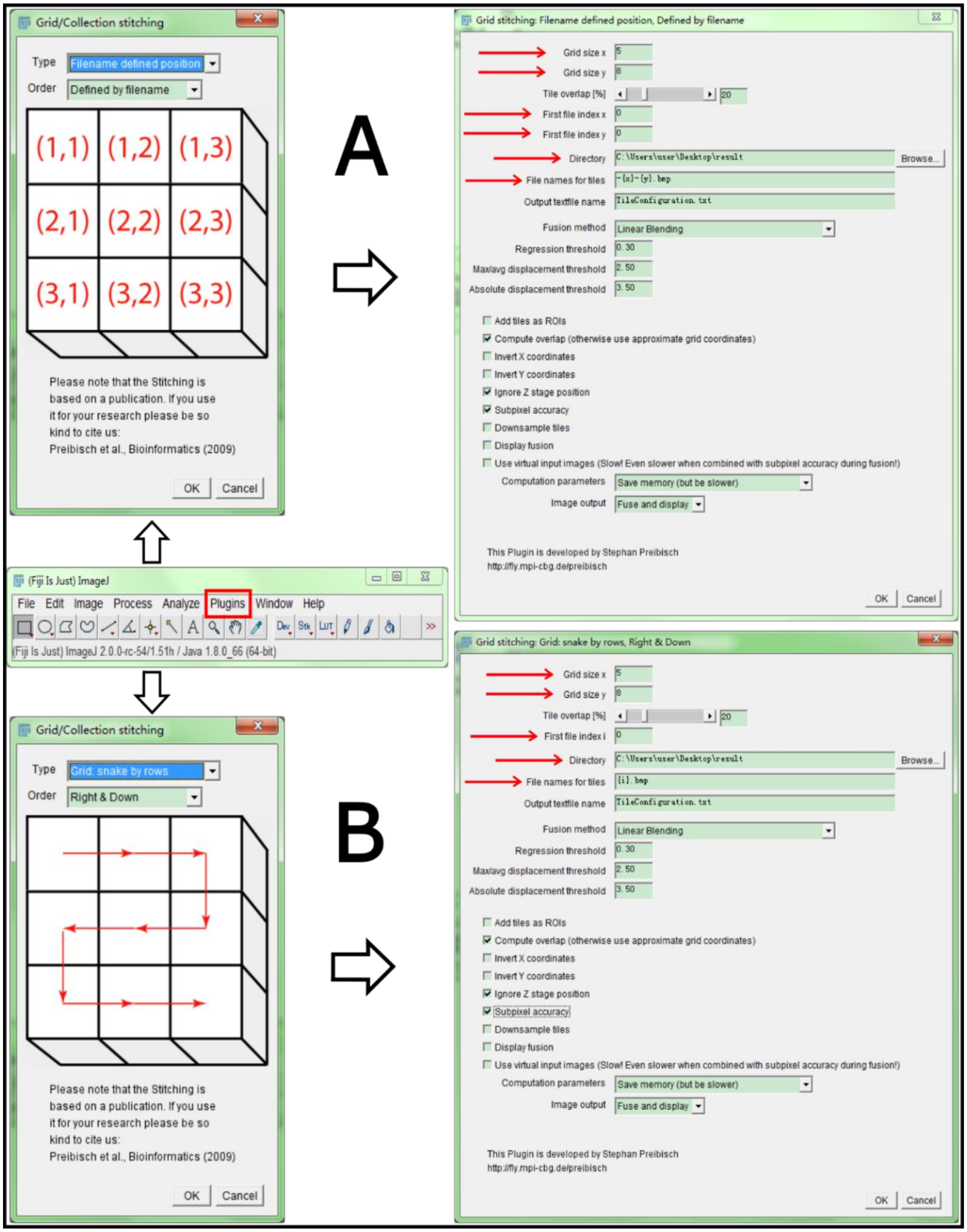

S11 Fig. Selection panel to mosaic images by Fiji

option, “Right & Down” is chosen. In the next dialog box, “Grid size x” and “Grid size y” are the values of “X times” and “Y times” from the “Square scanning” software. “First file index i” option should be set to “0”, “Directory” should be the location of the images, and the “File name for tiles” should be set as shown ({i}.bmp).

4.4. Software for USB camera

The exposure settings of the USB camera are controlled using a separate software ("Mind Vision 演示程序") that can be freely downloaded: http://www.mindvision.com.cn/rjxz/list_12.aspx?lcid=63. Although the language of this software is Chinese, translations of the critical settings are shown below in S12 Fig.

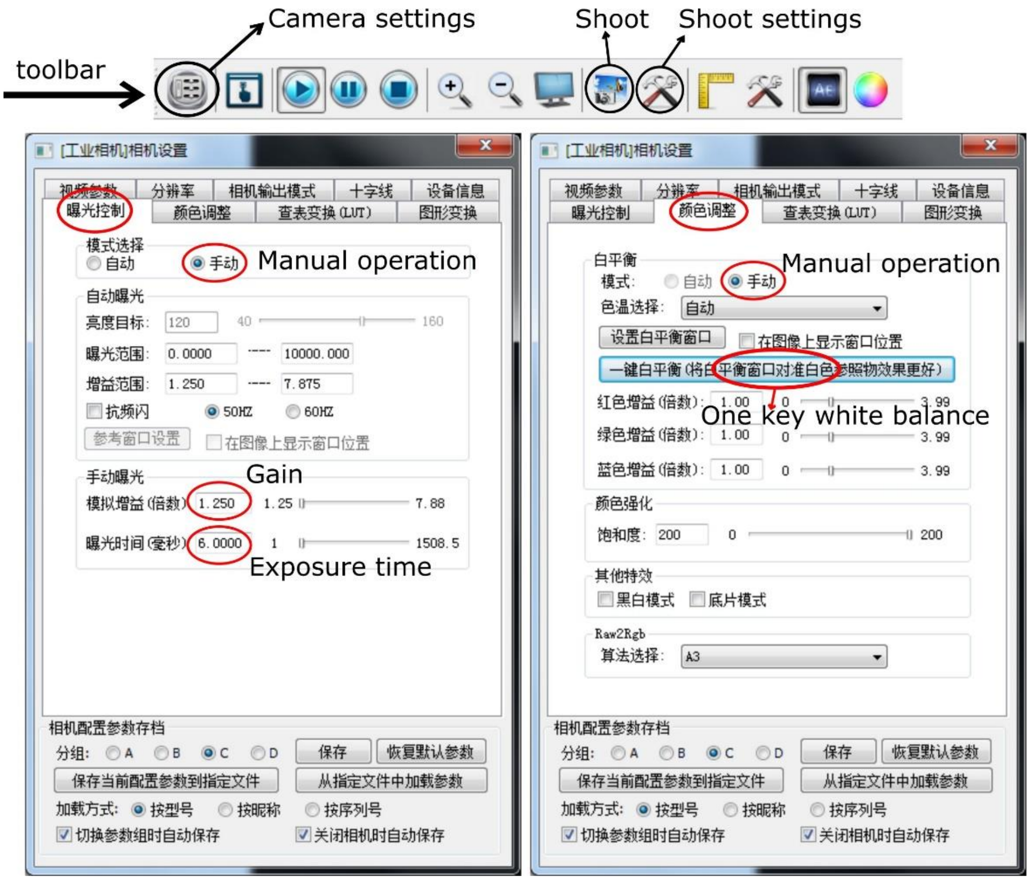

S12 Fig. Useful choices to use the camera control software

In the 'Camera setting', only two panels of 'exposure control' (S12 Fig left) and 'color adjustment' (S12 Fig right) are important. The settings that are labeled in red circles should be chosen. For bright field operation, Gain of 1.25 and Exposure time of 5.0 ms are suitable. For fluorescence images, depending on the sample, Gains of 5-10 and Exposure times of 400 ms or so are required, due to the much weaker emission. In order to reduce noise in the fluorescence images, red and blue gains should be set to zero.
